# Lentivirus-mediated overexpression of netrin-1/DCC co-expression promotes axonal regeneration and functional recovery in spinal cord injury via the inhibition of the NgR1-RhoA-ROCK signaling pathway

**DOI:** 10.1515/tnsci-2025-0365

**Published:** 2025-03-10

**Authors:** Meng-ling Zheng, Zheng Ma, Yuan-xia, Li-Juan Wang, Yan Fan, Cheng-An Feng, Jian-Ping Zhou, Zhong-Ming Li, Cheng-Xing Liu, Yan-Bin XiYang, Ying-Chun Ba

**Affiliations:** Department of Anatomy and Histology & Embryology, Faculty of Basic Medical Science, Kunming Medical University, KunMing, YunNan, China; Institute of Neuroscience, Kunming Medical University, KunMing, YunNan, China; Department of Orthopedics, People’s Hospital of Chenggong District, Kunming City, Yunnan, China; Department of Human Anatomy, Haiyuan College, Kunming Medical University, KunMing, YunNan, China

**Keywords:** spinal cord injury, lentivirus, axonal regeneration, Netrin-1, DCC, NgR1-RhoA-ROCK signaling pathway

## Abstract

Spinal cord injury (SCI) seriously affects the health of humans and quality of life, causing disabilities. Due to the ever-increasing traffic and cases of natural disasters, such as earthquakes, the incidence of SCI increases every year, thus causing a huge economic burden to society and patients. The lack of neurotrophic factors in the area affected by SCI and the presence of inhibitory factors for axonal regeneration are important reasons that make spinal cord regeneration and repair extremely difficult. Additionally, the correct projection of axons also plays an important role. As Netrin-1 is a signaling factor that guides axon growth, in this study, to determine whether Netrin-1 can promote axonal regeneration after binding to the receptor DCC following SCI, a Netrin-1/DCC co-expression recombinant lentiviral vector was constructed. This vector was used to assess the effect of Netrin-1 on the NgR1-RhoA-ROCK signaling pathway in an SCI model constructed in this study. Our results suggested that Netrin-1 exerts neuroprotective effects by inhibiting the NgR1-RhoA-ROCK signaling pathway after binding to its receptor DCC.

## 1 Introduction

Spinal cord injury (SCI) seriously affects health and decreases the quality of life by causing disabilities. The level of injury produces significant motor, sensory, and autonomic dysfunction that cannot be effectively cured [1]. The SCI process can be divided into primary injury and secondary injury [2,3]. Primary injury is mainly the destruction of axons and neurons [4]. Secondary SCI can be divided into several continuous stages: acute, subacute, subchronic, and chronic [5]. In the acute phase of secondary injury, primary traumatic injury triggers multiple pathophysiological processes, including neutrophil invasion, neuronal death, axon swelling, and blood–brain barrier permeability. In the subacute phase (7–14 days after SCI), injury and repair occur simultaneously. The main processes are macrophage infiltration, blood–brain barrier repair, edema resolution, and scar formation [6,7]. The subacute phase is considered to be the critical period for biotherapy. The spontaneous repair process after SCI is affected by many factors, such as neuroinflammation, fibrous scars, glial scarring, and myelin debris, the presence of axon growth inhibitors, low levels of injury signaling mechanisms, and reduced expression of regrowth-related genes, apoptosis, and failure of axon guidance [8].

Although there is no consistently effective cure for SCI, several therapeutic ideas or strategies have been proposed. These therapeutic ideas include comparing the differences in gene expression profiles between the regenerative peripheral nervous system (PNS) and the non-regenerative central nervous system (CNS) [9], neutralizing growth inhibitors in the environment of axonal regeneration [10,11,12], and comparing gene expression profiles between the regenerative PNS and the non-regenerative CNS, providing neurotrophic factors (NTFs) to promote axonal growth and guidance, neural stem cell transplantation [13,14], connecting spinal cord lesions with nerve growth scaffolds to restore the activity of residual fibers (including the repair of damaged myelin sheath), and increasing neuronal plasticity by stimulating the compensatory growth of intact fibers above and below the lesion. However, the growth of regenerating axons is often undirected and cannot be routed through the site of injury to restore connectivity with the appropriate distal target [15]. Therefore, axon regeneration and its guidance is one of the important goals in SCI research. The lack of NTFs after SCI and the presence of factors that inhibit axonal regeneration are the main reasons that make spinal cord regeneration very difficult. Many experimental and clinical studies have also shown that administering NTFs alone cannot lead to a satisfactory regeneration effect after central nerve injury [16]. Remon suggested that the correct projection of nerve axons is mediated by diffusible chemical molecules [17]. Four signaling factors are assumed to guide the growth of axons in the CNS; these include repulsion factors and attraction factors produced on the surface of nerve cells that act over short distances and diffusible chemorepellents and attractors that act over long distances. These signaling factors include netrins, semaphorins, slits, and ephrins, and the role of these guiding factors is often twofold, i.e., they can induce attraction and repulsion [18,19,20]. Netrin-1 was initially identified as a potent chemotactic molecule involved in axonal guidance and cell migration during embryonic development for maintaining axonal integrity in the CNS/PNS [21]. It is a bi-functional secreted chemotropic ligand. When Netrin-1 binds to its receptor deleted in colorectal cancer (DCC) dimer, it acts as a chemoattractant and promotes axon growth. However, after binding to another receptor, UNC-5 or DCC isomer, Netrin-1 acts as a chemorepellent and transmits signals to inhibit axon growth [22].

The Rho signaling pathway strongly influences neurons and regulates axonal regeneration. The proteins in this pathway are members of the Ras superfamily and can be divided into three subfamilies according to their sequences and functions, namely Rho, Rac, and Cdc42. Upregulation of the Rho signaling pathway caused by SCI seriously hinders axonal regeneration [23,24]. The main members of the Rho family include RhoA, RhoB, RhoC, etc. Among them, RhoA is an important factor leading to nerve regeneration disorders. Racl and Cdc42 can promote nerve regeneration [25]. The RhoA-ROCK signaling pathway consists of three key molecules: RhoA, Rock, and myosin phosphatase (MP). When the NgR-RhoA-Rock signaling pathway is blocked upstream, the expression of RhoA decreases. In the process associated with SCI repair, three major inhibitors were found to be associated with the RhoA signaling pathway, including neurite outgrowth inhibitor (Nogo), myelin-associated glycoprotein (MAG), and oligodendrocyte myelin glycoprotein (OMgP). The Nogo gene encodes three proteins, including Nogo-A, Nogo-B, and Nogo-C. Nogo-A is an integral membrane protein that is expressed mainly by oligodendrocytes and strongly inhibits axonal growth *in vivo* and *in vitro* [26]. Although Nogo-A, MAG, and OMgp are structurally different, they activate RhoA by binding to the common receptor NgR, which then transmits signals to ROCK, eventually leading to growth cone collapse [27]. Some studies have also found that after SCI, the RhoA signaling pathway not only inhibits axon regeneration but also promotes neuronal apoptosis in the injured area. Additionally, knocking down RhoA in animals promotes axon regeneration and neuronal survival [28]. Therefore, the NgR1-RhoA-ROCK pathway is an important target for inhibiting structural and functional repair of neurons and treating SCI repair. In non-neuronal and neuroblastoma cells, DCC can activate Rac1 and Cdc42 to induce cytoskeletal reorganization and axonal attraction signaling. Rac1 and Cdc42 play key roles in DCC-induced axonal growth during embryonic neural development. Also, inhibition of RhoA and its downstream effector Rock can promote DCC-induced axonal growth [29]. When the growth cone grows toward the inducing signal, the activity of Cdc42 and Rac can exceed that of the Rho pathway. The repulsive signal of a growth cone is shown by the activation of the Rho pathway at the cost of the Rac pathway [30], and the activation of Rho causes filopodia retraction, leading to the collapse of the growth cone [31].

In conclusion, netrin-1 has a bidirectional effect: binding to its receptor DCC dimer mediates an attractive signal to axon growth cones and promotes axon growth while binding to another receptor UNC-5 or DCC isoform conveys an inhibitory signal to axon growth. The above studies are basically limited to neural development, but whether Netrin-1 can better promote axon regeneration and regulate the NgR1-RhoA-ROCK signaling pathway by binding to its receptor DCC after nerve injury has not been reported. Whether Netrin-1 promotes axon regeneration by inhibiting the NgR1-RhoA-ROCK signaling pathway after binding to its receptor DCC has not been reported. Understanding these mechanisms will be of great help to overcome the problem of repair after SCI in clinical practice as soon as possible, and will open up a new way for clinical treatment of SCI.

## 2 Materials and methods

### 2.1 Animals and groups

Adult Sprague–Dawley (SD) rats (200–250 g), provided by the Laboratory Animal Center of Kunming Medical University, were used in this study. All rats had free access to food and water in plastic cages with a 12h/12h light/dark cycle. The animals were randomly assigned to the following groups: sham group, SCI group, Netrin-1-ORF-NC group, Netrin-1-ORF group, Netrin-1-si-NC group, Netrin-1-si group, DCC-ORF-NC group, DCC-ORF group, DCC-si-NC group, DCC-si group, (Netrin-1/DCC)-ORF-NC group, (Netrin-1/DCC)-ORF group, (Netrin-1/DCC)-si-NC group, and (Netrin-1/DCC)-si group.

### 2.2 Construction of Netrin-1 and DCC lentiviruses

Recombinant lentiviruses expressing Netrin-1 interfering RNA/scramble RNA (NC), Netrin-1 overexpression/scramble RNA (NC), and DCC interfering RNA (siRNA)/scramble RNA (NC) were constructed by Jikai (Shanghai, China). Among them, Netrin-1 overexpression exhibited red fluorescence, Netrin-1 interference exhibited green fluorescence, DCC overexpression exhibited green fluorescence, and DCC interference exhibited red fluorescence.

### 2.3 SCT surgical procedures

The anesthetized rats were fixed in the prone position on a brain stereotaxic instrument. The spinal canal was occluded, after which T9, T10, and T11 spinal cords were exposed, the spinal cord was fully transected at the T10 level, and Netrin-1 and DCC lentiviruses (1 µL, 1 × 10^10^ pfu/mL, *n* = 6) were meanwhile injected locally using a microinjector at the site of SCI; three injection sites were selected at each of the upper and lower ends of the transected region, each with 5 µL. Rats were euthanized if they exhibited very abnormal behavior after SCI, such as 40% weight loss, self-mutilation of lower extremities or abdomen resulting in inability to urinate, difficulty in breathing, or very painful phenomena.

### 2.4 Basso–Beattie–Bresnahan score

Three researchers performed behavioral scoring for each group of rats on days 1, 7, 14, 28, 42, and 63 after total spinal cord transection. To avoid subjective bias in the scoring process, the mean value of the scores was obtained after they were recorded by the three individuals using a double-blind method; these values were used for statistical analyses.

### 2.5 Tissue harvest

On days 1, 7, 14, and 28 after the operation, for RT-PCR and WB experiments, the spinal cord tissues were removed and stored in liquid nitrogen quickly. For immunofluorescence staining, the samples were harvested after intracardiac perfusion with 0.9% physiological saline followed by 4% paraformaldehyde in 0.1 M ice-cold phosphate buffer (pH 7.4).

### 2.6 Immunofluorescence staining

Harvested spinal cord tissues were dehydrated in a 4% paraformaldehyde solution overnight. Then, the tissues were sliced into thin sections. The paraffin-embedded spinal cord tissues were dewaxed and hydrated with gradient ethanol. The sections were washed thrice in 0.01 M phosphate-buffered saline (PBS) and incubated with 5% goat serum for 30 min at room temperature to block non-specific binding, followed by incubation with primary antibodies at 4°C. The primary antibodies used were specific to SYP (Proteintech, 17785-1-AP, rabbit, 1:400) and GAP-43 (Abcam, ab75810, rabbit, 1:500). Next, the sections were washed with PBS three times for 5 min each and incubated in fluorescence-labeled secondary antibodies Alexa Fluor^®^ 488 (Bioss, Goat against rabbit, 1:200) and Alexa Fluor^®^ 594 (Bioss, Goat against rabbit, 1:200) at 37°C in the dark for 1 h. The nuclei of the cells were stained by adding 4′,6-diamidino-2-phenylindole (DAPI, Solarbio) containing an anti-fluorescence quencher, after which the sections were sealed with coverslips. The images of the samples were captured using a fluorescence microscope. The cells in these images were counted using the ImageJ software programs, and the axon length was examined.

### 2.7 TUNEL staining

TUNEL staining was performed using a one-step TUNEL *in situ* apoptosis detection kit (KeyGEN BioTECH, China). Initially, cell samples were immersed in 1% Triton X-100 permeabilizing solution for 5 min at ambient temperature. Next, a proteinase K working solution was added dropwise to the paraffin sections of spinal cord tissue for 30 min at 37°C. Staining was performed using a TdT enzyme reaction solution and streptavidin–fluorescein reagent. Finally, the tissue samples were stained with DAPI containing an anti-fluorescence quencher for 5 min at room temperature. For each sample, images were captured from five different areas using an inverted fluorescence microscope. The number of cells was counted using the ImageJ software, and the apoptosis rate was calculated by determining the total number of TUNEL-positive neurons and DAPI-labeled cells.

### 2.8 RT-qPCR assay

On days 1, 7, 14, and 28 after SCI, the areas 0.5 cm above and below the site of the SCI were examined. The tissue was collected and homogenized using a tissue grinder. Primers for Netrin-1, DCC, NgR, RhoA, Rock1, and Rock2 were designed using the Primer 5.0 software. Total RNA was extracted from tissues using RNAiso™ Plus (Takara, Japan). Then, cDNA was synthesized using a reverse transcription kit (Vazyme, China). The Taq Pro Universal SYBR qPCR Master Mix (Vazyme, China) and RT-qPCR system (Thermo Fisher Scientific, DE) were used to measure the total cDNA transcribed. The β-actin gene was used as an endogenous reference. The fluorescence values were recorded from each reaction, and real-time PCR monitoring of RNA was performed to achieve relative, absolute, and qualitative analysis of genes. The final threshold cycle [32] value was determined by recording the intersection value of the baseline and the curve, and then, the expression of each factor and its difference was processed and analyzed using Microsoft Excel and SPSS. The 2^−△△*C*^_t_ method was used to analyze the data.

### 2.9 Western blotting analysis

The experiments were performed on days 7, 14, and 28 post-SCI to examine tissue 0.5 cm above and below the site of the SCI. The tissue was homogenized using a tissue grinder. The rest of the procedure was performed as described above. As the stock of primary antibodies used in the above experiment was over, we used antibodies of other brands, and the information on primary antibodies is as follows: Netrin-1 antibody (Abcam, Rabbit, 1:1,000, item no. ab126729), DCC antibody (Abcam, Rabbit, 1:1,000, item no. ab273570), Nogo Receptor antibody (Abcam, Rabbit, 1:10,000, item no. ab184556), RhoA antibody (Abcam, Rabbit, 1:5,000, item no. ab187027), and ROCK1/2 antibody (Abcam, Rabbit, 1:2,000, item no. ab45171). The samples were incubated with these antibodies overnight at 4°C for 16–18 h; β-actin (Abbkine, Mouse, 1:2,000) was used as a control. After washing with T-BST, the samples were incubated with secondary antibodies (HRP, goat anti-rabbit IgG, 1:5,000; HRP, goat anti-mouse IgG, 1:5,000) for 60 min, and then, they were washed three times with T-BST every 10 min. The strips were placed on a gel imager (BIO-RAD, USA), and the signal was developed in a dark room using an enhanced chemiluminescence reagent (Pierce, Rockford, USA). The bands developed were normalized and quantified using the ImageJ software to determine band intensity.

### 2.10 Statistical analysis

All data were analyzed using SPSS24.0 (IBM Corporation, NY, USA). The results are expressed as the mean ± standard deviation (SD). The differences in parameters between the two groups were determined by Independent samples *t*-tests; the differences were considered to be statistically significant at *p* < 0.05. The differences in parameters among multiple groups were statistically analyzed using a completely randomized design and by conducting one-way ANOVA (pairwise comparison of multiple sample means). The homogeneity of variance test probability, *p* < 0.05, was considered as uneven variance, Dunnett’s T3 test was used, and *p* > 0.05 was considered homogeneity of variance, and the LSD test was used.


**Ethical approval:** The research related to animals’ use complied with all the relevant national regulations and institutional policies for the care and use of animals. Protocols for animal experiments were approved by the Animal Experimental Ethics Committee of the Kunming medical University compliance with the National Institutes of Health guidelines for the care and use of laboratory animals. All animals were cared for in strict accordance with the Guide for the Care and Use of Laboratory Animals (NIH Publication No. 85-23, revised 1996), and the experimental design was approved by the Ethics Committee of Kunming Medical University.

## 3 Results

### 3.1 Construction of the rat SCI model

**Figure 1:**
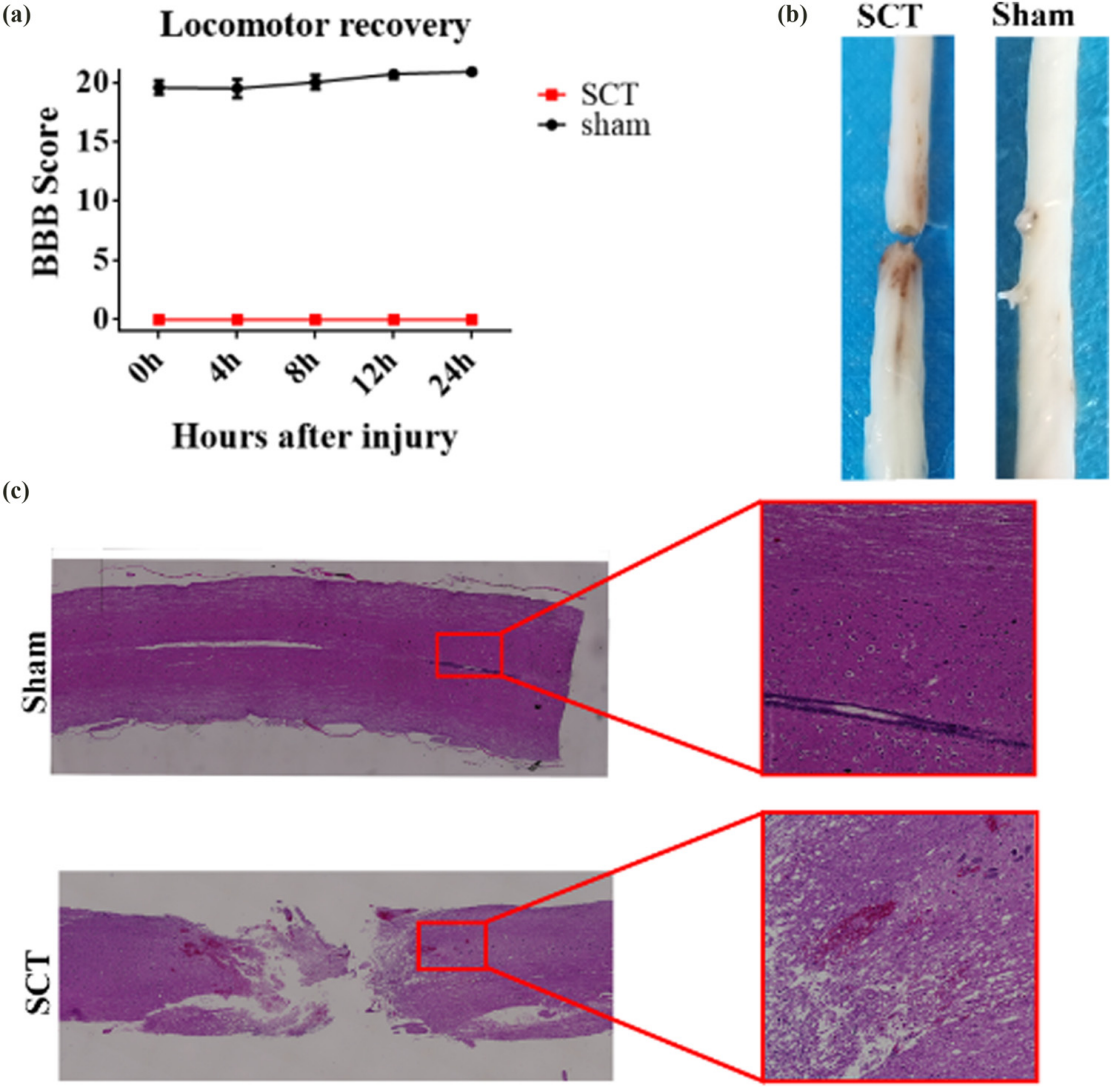
Assessment of SCI: (a) The corresponding BBB scores of rats in the Sham and SCT groups were quantified (*n* = 6); (b) morphology of the spinal cord of rats in Sham and SCT groups; and (c) HE staining was used to observe the morphological changes in the spinal cord in the Sham group and SCI group.

To examine the effect of Netrin-1 and DCC on functional recovery in SCI rats after binding, we used the full SCT rat model. Immediately after complete SCT was performed at the T10 level, the hind limbs of the rats were paralyzed. To assess the motor function of the hind limbs, we performed BBB tests (BBB motor rating scale) 0, 4, 8, 12, and 24 h after SCI and the BBB score was found to be 0 (Figure 1a). The transection injury was visible to the naked eye (Figure 1b). H & E-stained sections showed that the Sham group exhibited normal neuronal morphology in the spinal cord, whereas most cells in the SCT group showed irregular morphology (Figure 1c).

### 3.2 SCI injury decreases the expression of Netrin-1 in spinal cord tissue

**Figure 2:**
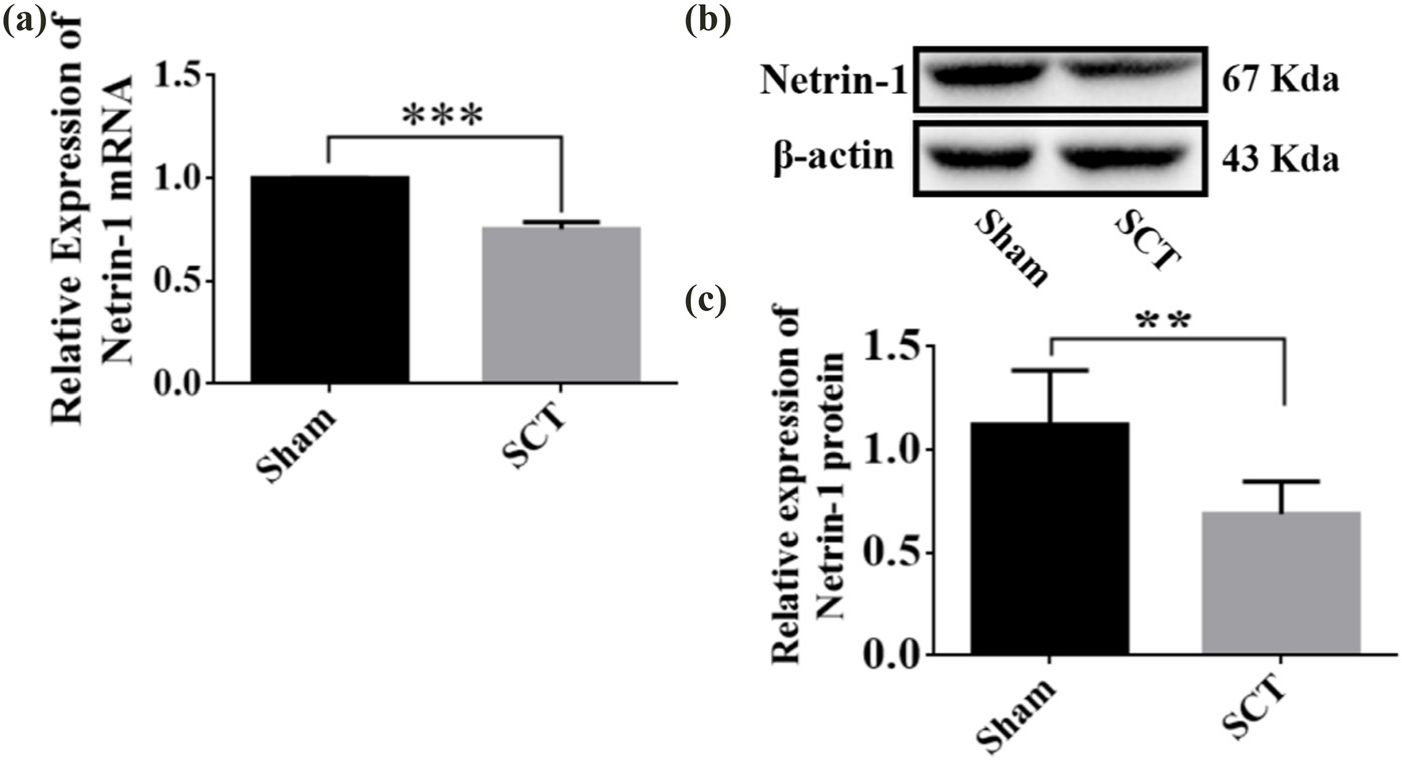
Changes in mRNA and protein expression of Netrin-1 1 day after SCI. (a) Histogram of mRNA expression of Netrin-1 in the Sham group and the SCT group. ***p < 0.001, statistically significant; (b) representative immunoblots of Netrin-1 and β-actin in spinal cord tissues from Sham and SCT groups after SCI, from left to right, respectively; and (c) protein quantification calibrated by β-actin, relative expression of Netrin-1 is Netrin-1/β-actin, for each group. The sample size was *n* = 6. All results were statistically significant as evaluated using independent *t*-tests, **p < 0.01.

One day after SCI, the expression of Netrin-1 mRNA was found to be significantly lower in the SCI group compared to that in the Sham group, as determined by PCR (Figure 2a). Next, the results of WB experiments showed that the relative expression of Netrin-1 protein in spinal cord tissue was also significantly lower in the SCI group compared to that in the Sham group (Figure 2b and c). These results suggested that SCI can stimulate a decrease in Netrin-1 expression in spinal cord tissues. Therefore, Netrin-1 might play a role after SCI.

### 3.3 Temporal expression levels of the Netrin-1 lentivirus in the spinal cord after SCI

**Figure 3:**
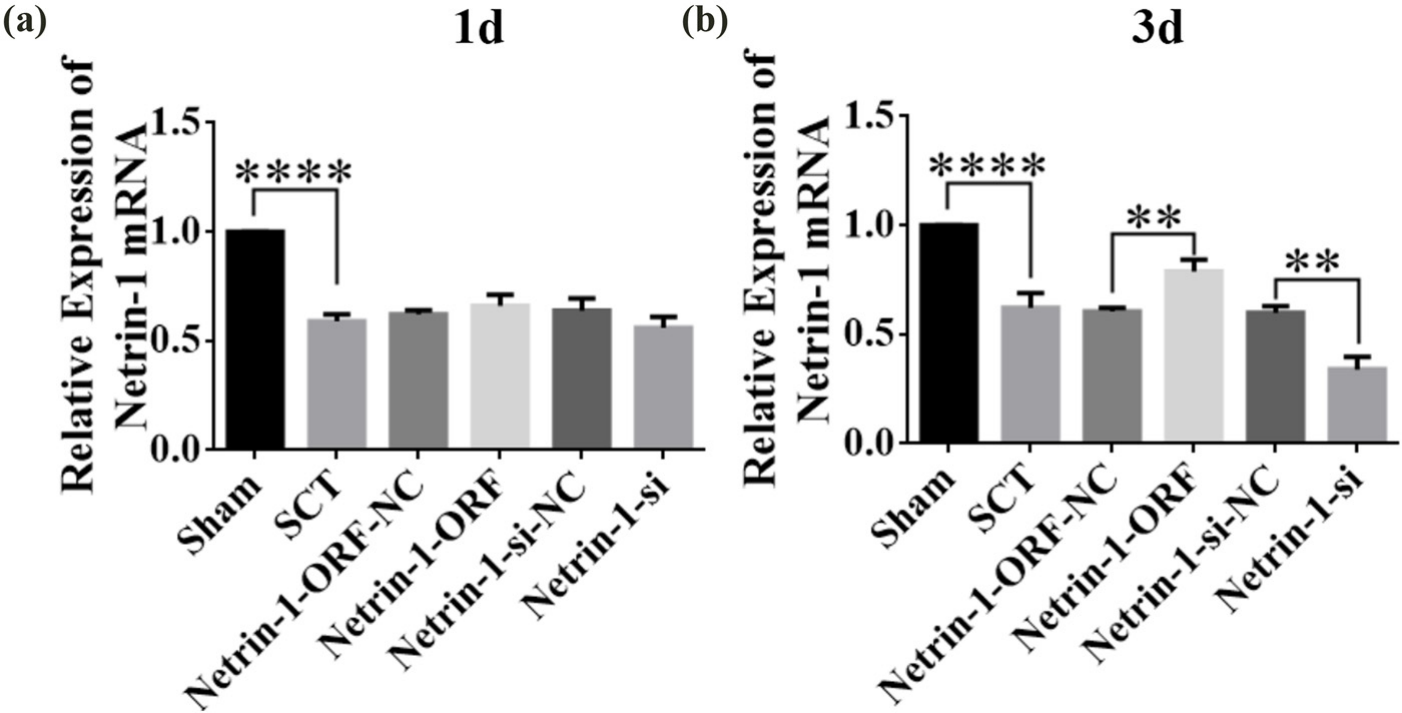
Netrin-1 overexpression/low expression lentivirus at 1 and 3 days after SCI. (a) 1 day after SCI, horizontal coordinates: Sham group, SCT group, Netrin-1-ORF-NC group, Netrin-1-ORF group, Netrin-1-si-NC group, and Netrin-1-si group; vertical coordinates indicate the relative mRNA expression of Netrin-1. (b) 3 days after SCI, horizontal coordinates: Sham group, SCT group, Netrin-1-ORF-NC group, Netrin-1-ORF group, Netrin-1-si-NC group, and Netrin-1-si group; vertical coordinates indicate the relative expression of mRNA of Netrin-1. Results are expressed as mean ± SD. Ordinary one-way ANOVA and LSD tests were performed: **p < 0.01, ****p < 0.0001.

Netrin-1 overexpression/low expression lentivirus was injected into the spinal cord immediately after spinal cord transection at the upper and lower ends of the transection site. One day after SCI, the results of PCR assays showed that the expression of Netrin-1 was significantly lower compared to that in the Sham group (Figure 3a). Although the overexpression and low expression groups showed higher and lower levels of expression of Netrin-1 compared to the control group, the differences between the groups were not statistically significant (Figure 3a). The low-expression group showed a lower level of expression of Netrin-1 than the control group, but the difference between the groups was not statistically significant (Figure 3a). The expression of Netrin-1 in the overexpression and low-expression groups was higher and lower than its expression in the control group, respectively, but the differences were not statistically significant (Figure 3a). Three days after SCI, the results of PCR assays showed that the expression of Netrin-1 was significantly lower than that in the Sham group (Figure 3b). The expression of Netrin-1 was significantly higher in the overexpression group compared to that in the control group (Figure 3b). The expression of Netrin-1 was significantly lower in the low-expression group compared to that in the control group (Figure 3b). These results indicated that Netrin-1 overexpression/low-expression lentivirus was not fully expressed in the spinal cord tissue 1 day after SCI, and its expression started 3 days after SCI. We waited till Netrin-1 was expressed in spinal cord tissues before testing its role after SCI was induced; therefore, the function of Netrin-1 was tested in the subacute and chronic phases after inducing SCI.

### 3.4 Netrin-1 affects the morphology and viability of neurons in spinal cord tissue and rat motor function

**Figure 4:**
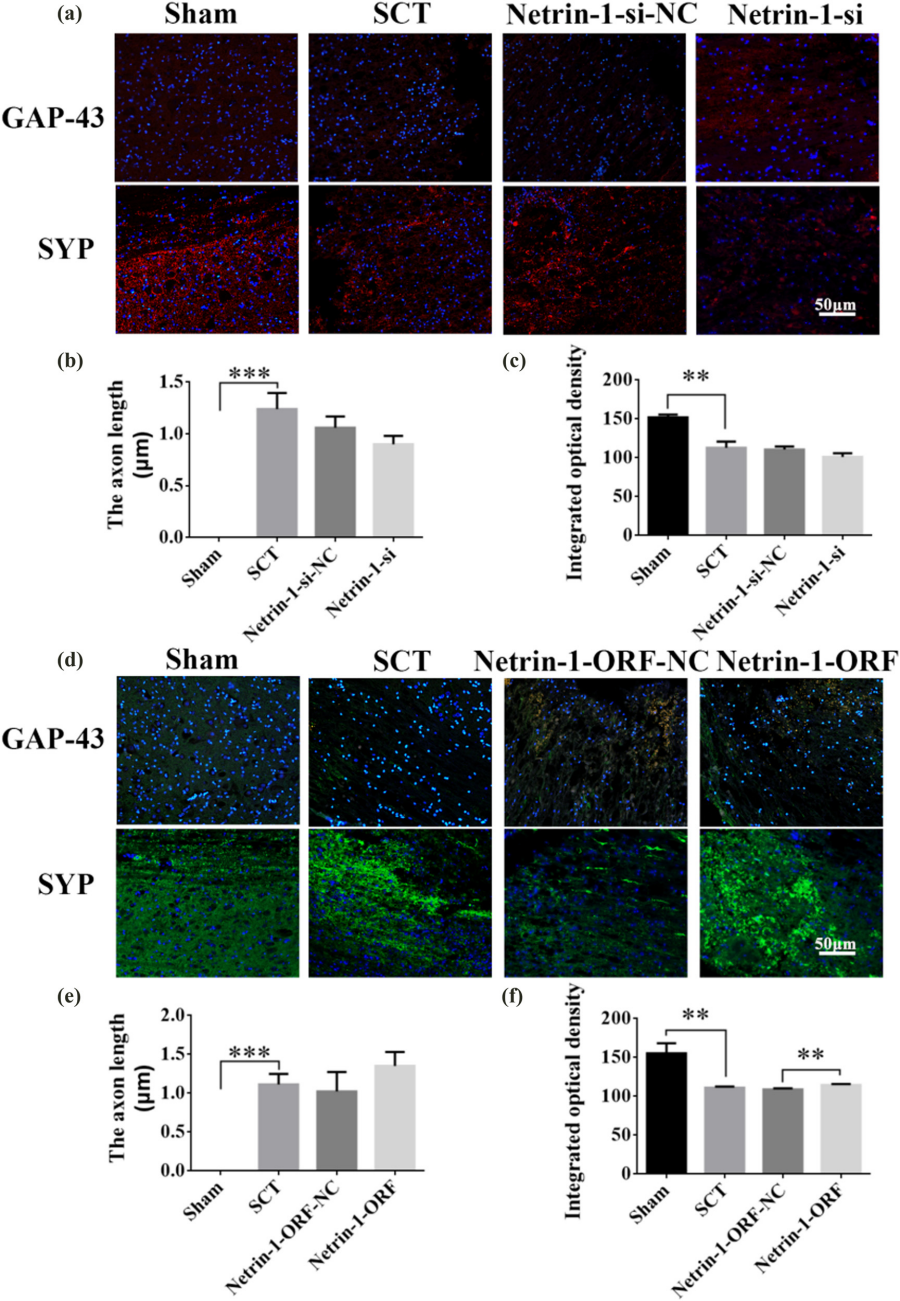
Histological analysis of Netrin-1 expression increased/decreased after 1 day post-SCI. (a) Immunohistochemical staining was used to determine GAP43 (neurospecific protein associated with synaptic development and neuronal regeneration, red) and SYP (synaptophysin, red) expression. DAPI: nuclear marker (blue), bar = 50 µm. (b) and (c) Quantitative statistics of GAP43-positive nascent axons and fluorescence intensity of SYP-positive in Sham, SCT, Netrin-1-si-NC, and Netrin-1-si groups. *n* = 6. (d) Immunohistochemical staining was used to determine GAP43 (green) and SYP (green) expression. DAPI (blue), bar = 50 µm. (e) and (f) Quantitative statistics of GAP43-positive nascent axons and fluorescence intensity of SYP-positive in Sham, SCT, Netrin-1-si-NC, and Netrin-1-si groups. *n* = 6. Results are expressed as mean ± SD. Ordinary one-way ANOVA and LSD tests were performed: **p < 0.01, ***p < 0.001, and ****p < 0.0001.

**Figure 5:**
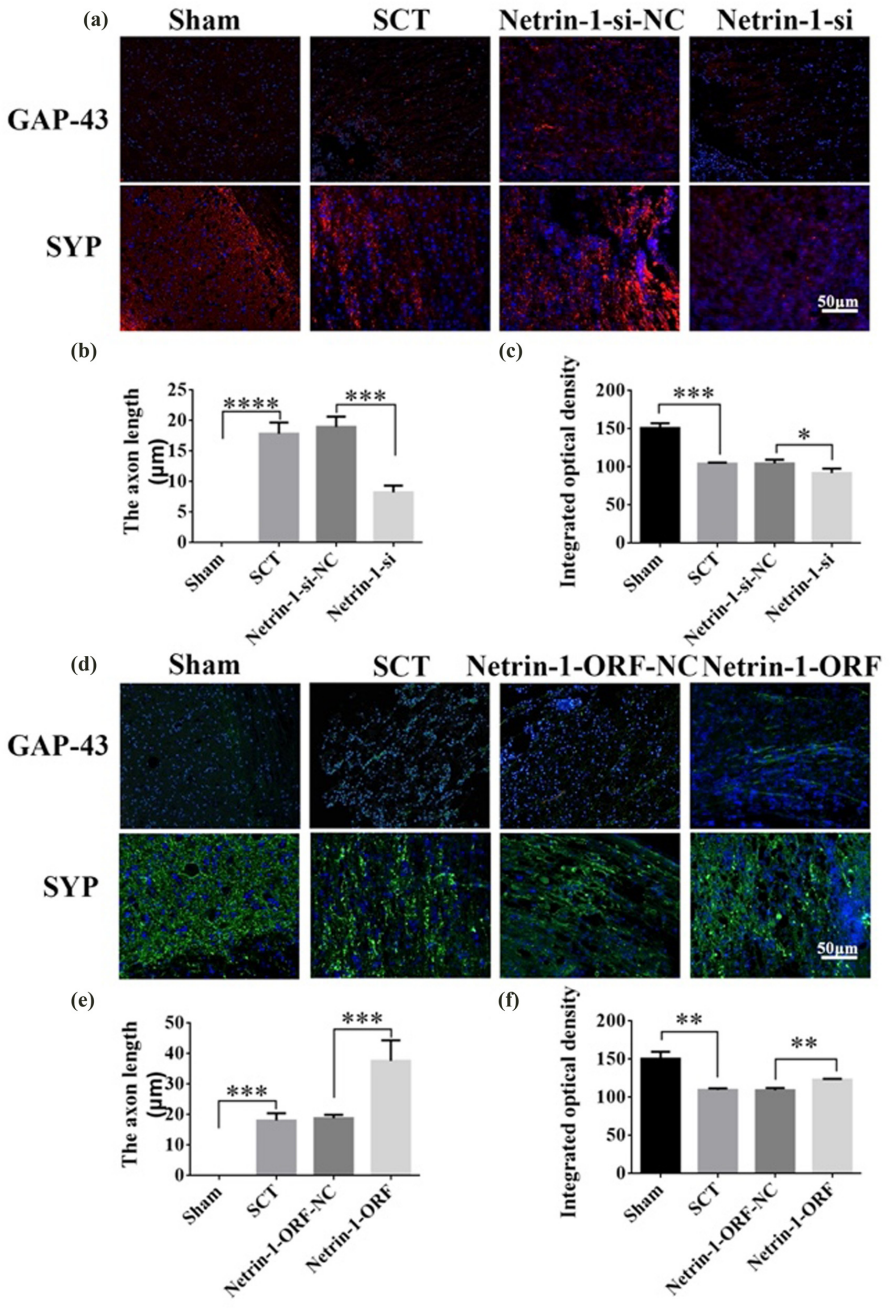
Histological analysis of Netrin-1 expression increased/decreased after 7 days post-SCI. (a) Immunohistochemical staining was used to determine GAP43 (neurospecific protein associated with synaptic development and neuronal regeneration, red) and SYP (synaptophysin, red) expression. DAPI: nuclear marker (blue), bar = 50 µm. (b) and (c) Quantitative statistics of GAP43-positive nascent axons and fluorescence intensity of SYP-positive in Sham, SCT, Netrin-1-si-NC, and Netrin-1-si groups. *n* = 6. (d) Immunohistochemical staining was used to determine GAP43 (green) and SYP (green) expression. DAPI (blue), bar = 50 µm. (e) and (f) Quantitative statistics of GAP43-positive nascent axons and fluorescence intensity of SYP-positive in Sham, SCT, Netrin-1-si-NC, and Netrin-1-si groups. *n* = 6. Results are expressed as mean ± SD. Ordinary one-way ANOVA and LSD tests were performed: **p < 0.01, ***p < 0.001, and ****p < 0.0001.

**Figure 6:**
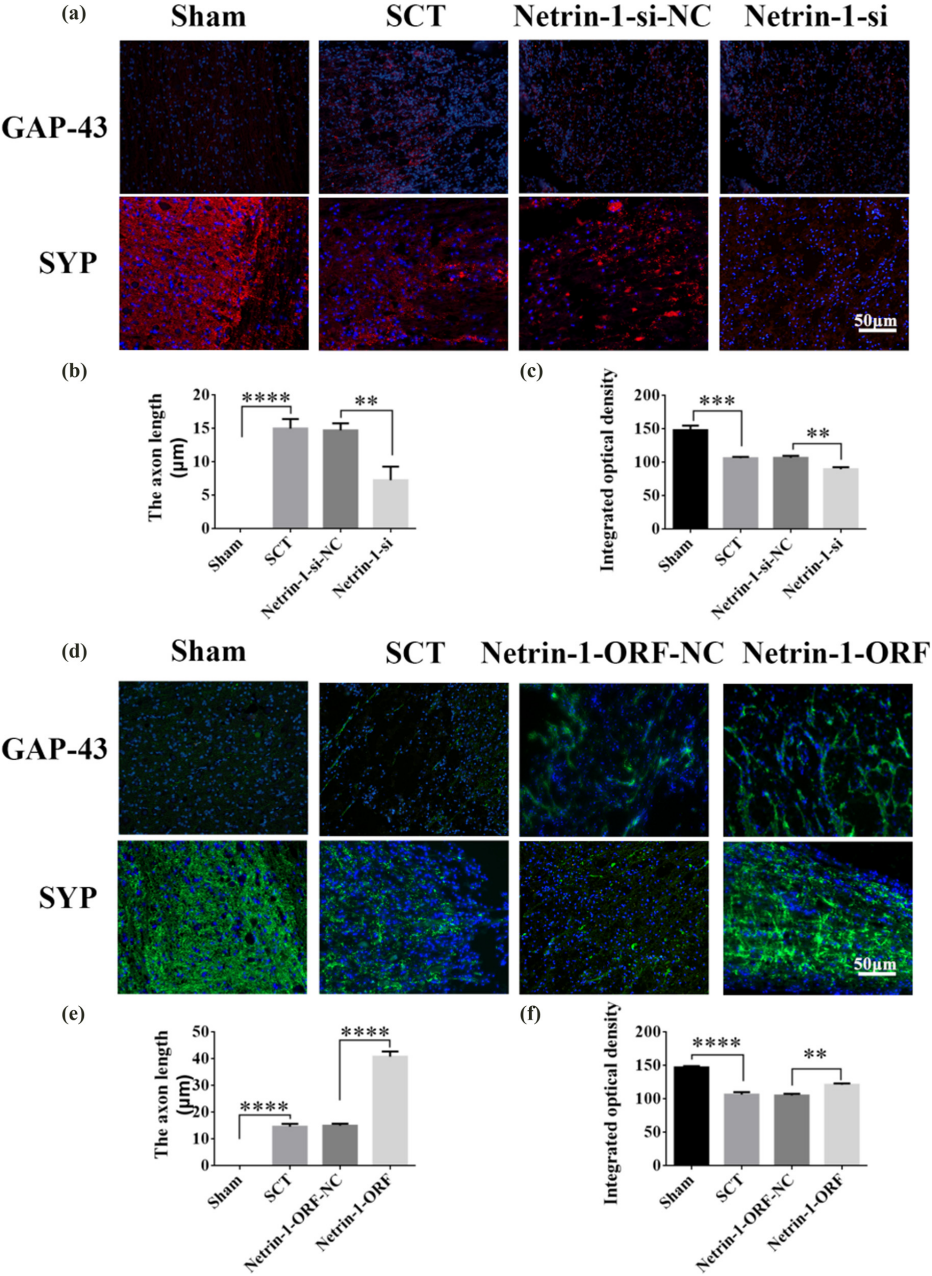
Histological analysis of Netrin-1 expression increased/decreased after 14 days post-SCI. (a) Immunohistochemical staining was used to determine GAP43 (neurospecific protein associated with synaptic development and neuronal regeneration, red) and SYP (synaptophysin, red) expression. DAPI: nuclear marker (blue), bar = 50 µm. (b) and (c) Quantitative statistics of GAP43-positive nascent axons and fluorescence intensity of SYP-positive in Sham, SCT, Netrin-1-si-NC, and Netrin-1-si groups. *n* = 6. (d) Immunohistochemical staining was used to determine GAP43 (green) and SYP (green) expression. DAPI (blue), bar = 50 µm. (e) and (f) Quantitative statistics of GAP43-positive nascent axons and fluorescence intensity of SYP-positive in Sham, SCT, Netrin-1-si-NC, and Netrin-1-si groups. *n* = 6. Results are expressed as mean ± SD. Ordinary one-way ANOVA and LSD tests were performed: **p < 0.01, ***p < 0.001, ****p < 0.0001.

**Figure 7:**
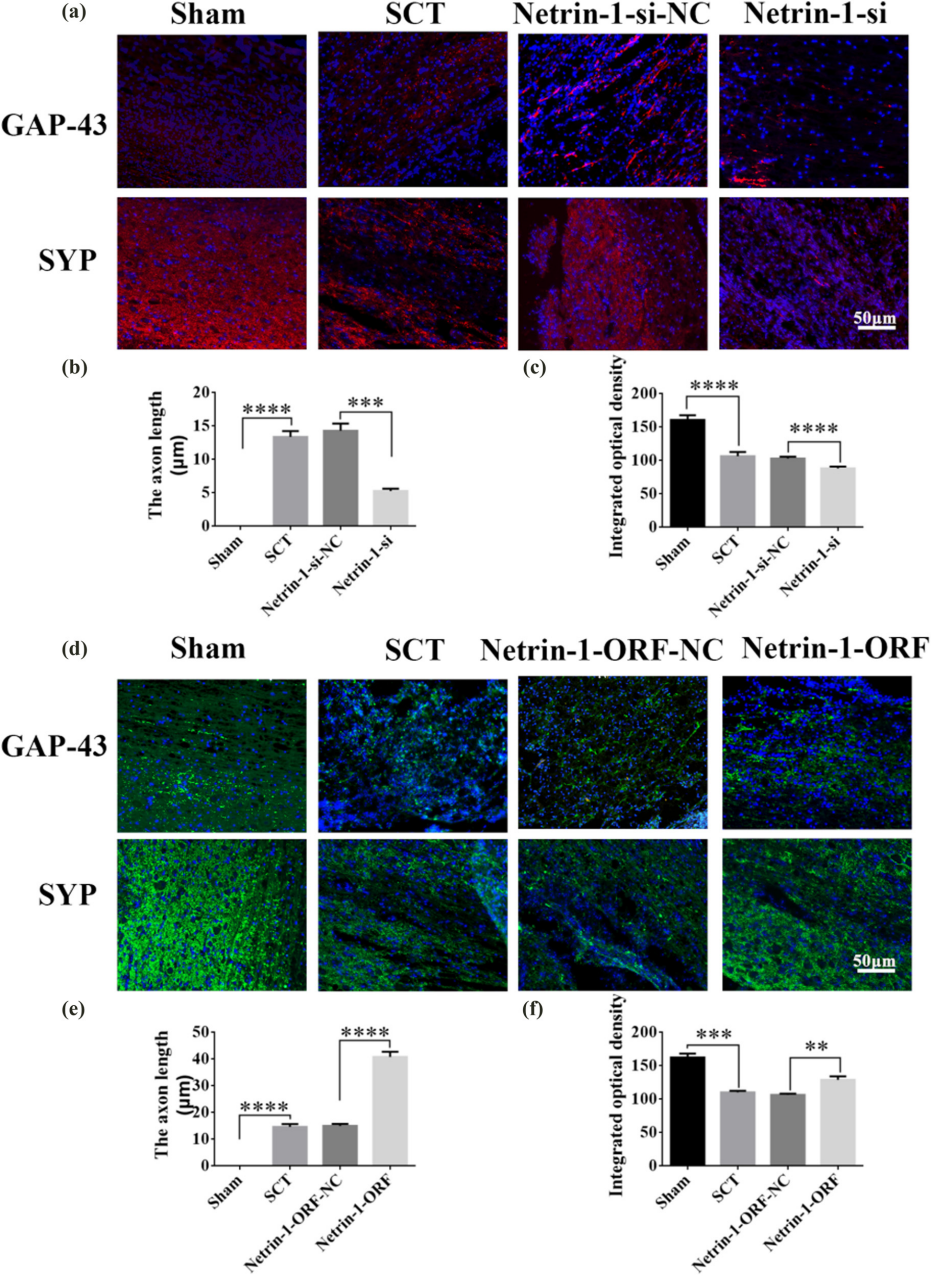
Histological analysis of Netrin-1 expression increased/decreased after 28 days post-SCI. (a) Immunohistochemical staining was used to determine GAP43 (neurospecific protein associated with synaptic development and neuronal regeneration, red) and SYP (synaptophysin, red) expression. DAPI: nuclear marker (blue), bar = 50 µm. (b) and (c) Quantitative statistics of GAP43-positive nascent axons and fluorescence intensity of SYP-positive in Sham, SCT, Netrin-1-si-NC, and Netrin-1-si groups. *n* = 6. (d) Immunohistochemical staining was used to determine GAP43 (green) and SYP (green) expression. DAPI (blue), bar = 50 µm. (e) and (f) Quantitative statistics of GAP43-positive nascent axons and fluorescence intensity of SYP-positive in Sham, SCT, Netrin-1-si-NC, and Netrin-1-si groups. *n* = 6. Results are expressed as mean ± SD. Ordinary one-way ANOVA and LSD tests were performed: **p < 0.01, ***p < 0.001, and ****p < 0.0001.

**Figure 8:**
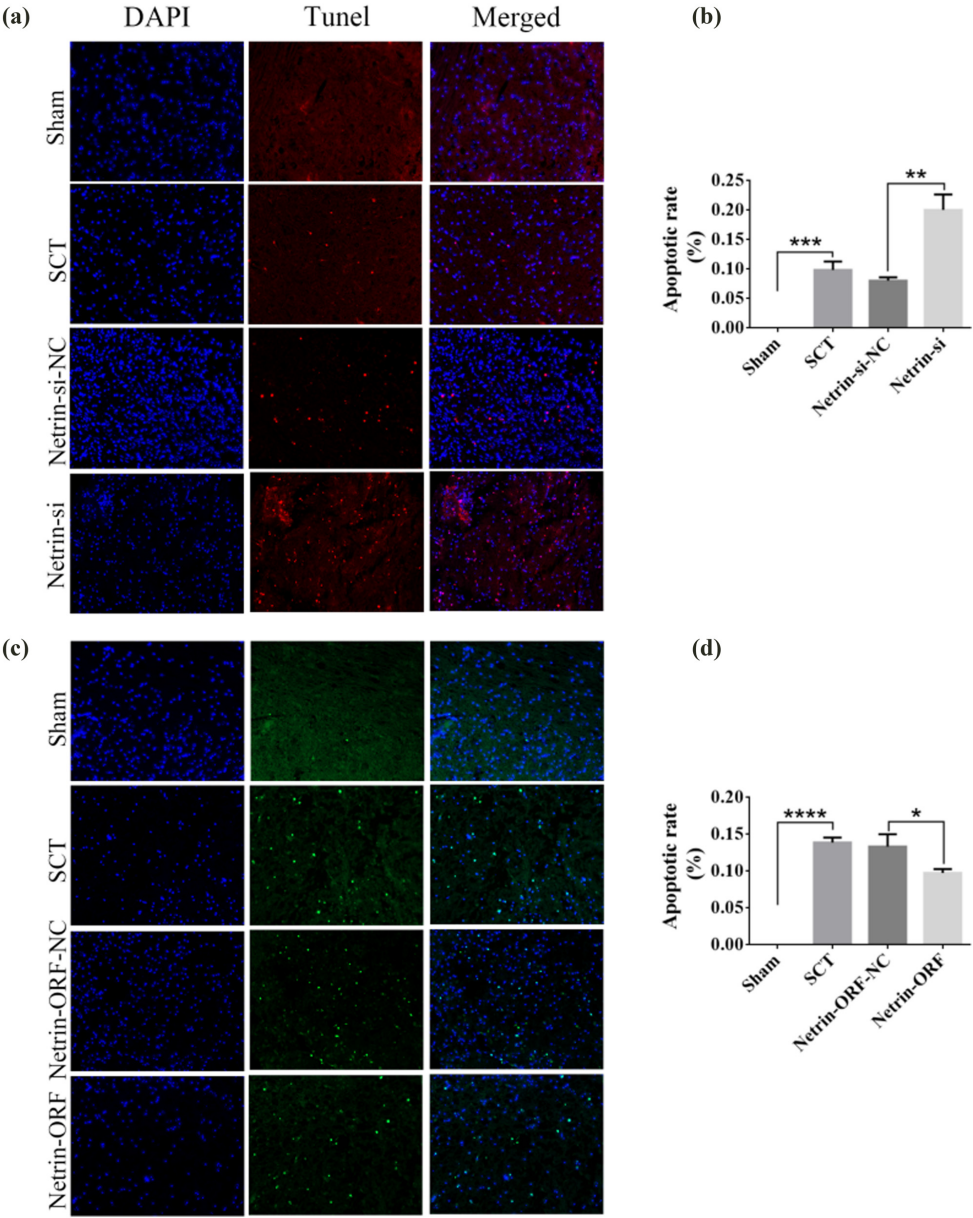
Tunel analysis of Netrin-1 expression increased/decreased after 7 days post-SCI. (a) DAPI (blue) indicates nuclei, and Tunel (red) indicates apoptosis. From left to right: DAPI, Tunel, TUNEL/DAPI Merged (fusion plot); from top to bottom, Sham group, SCT group, Netrin-1-si-NC group, and Netrin-1-si group under SCT conditions, bar = 50 μm. (b) Analysis of Sham, SCT, Netrin -1-si-NC, and Netrin-1-si groups for quantitative statistics of apoptosis. *n* = 6. (c) DAPI (blue) indicates nuclei, and Tunel (green) indicates apoptosis. From left to right: DAPI, Tunel, TUNEL/DAPI Merged (fusion plot); from top to bottom, Sham group, SCT group, Netrin-1-ORF-NC group under SCT conditions, and Netrin-1-ORF group; images are 200×, bar = 50 μm. (d) Quantitative statistics of apoptosis in Sham, SCT, Netrin-1-ORF-NC, and Netrin-1-ORF groups were statistically analyzed for apoptosis rate. *n* = 6. Results are expressed as mean ± SD. Ordinary one-way ANOVA and LSD tests were performed: *p < 0.05, **p < 0.01, ***p < 0.001, and ****p < 0.0001.

**Figure 9:**
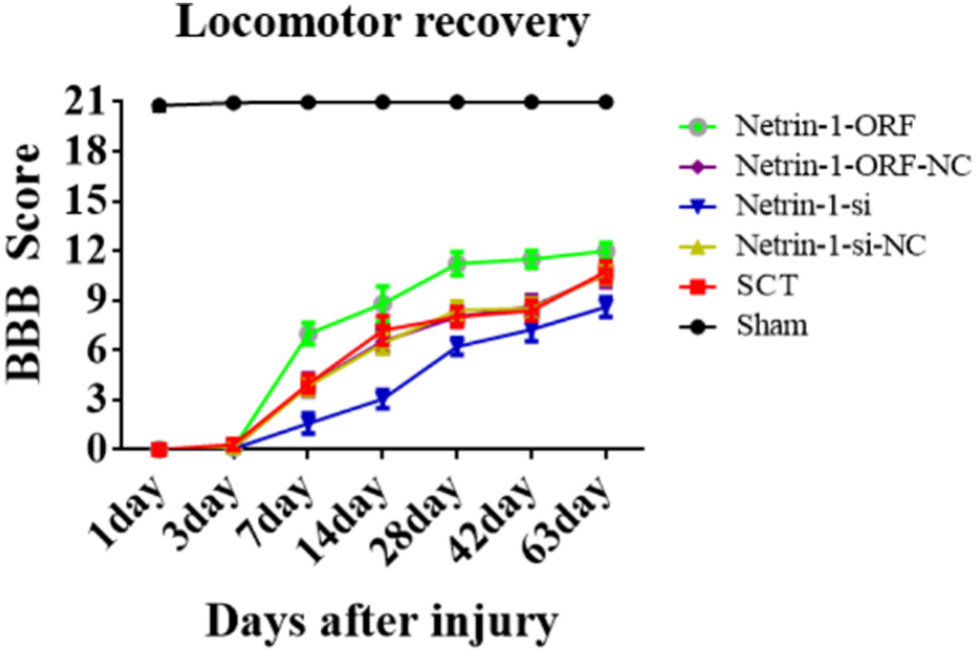
Behavioral analysis after increase/decrease of Netrin-1 expression. The abscissa represents 1, 3, 7, 14, 28, 42, and 63 days, and the ordinate represents the BBB score.

On days 1, 7, 14, and 28 after SCI, we examined the morphological changes in GAP43 (neuromodulin, an axon membrane protein, is a neuron-specific protein involved in extracellular growth, synapse formation, and nerve cell regeneration; it is expressed at high levels during neuronal development and regeneration) and SYP cells (Figure 4a and d). We also quantified the length of the axon of newborn neurons and the fluorescence intensity of positive cells. The results indicated that the axons started becoming nascent 1 day after SCT, and since Netrin-1 overexpression/low-expression lentivirus was not yet fully expressed in spinal cord tissues, an increase or decrease in Netrin-1 expression did not significantly affect the treatment of axon regeneration after SCI (Figure 4). Also, an increase in Netrin-1 expression promoted axonal regeneration in the subacute and chronic phases of SCI (on days 1, 7, 14, and 28 days after SCI) (Figures 5–7). As the role of Netrin-1 in promoting axonal regeneration was detected starting on day 7 after SCI, we examined the morphological changes in Tunel cells 7 days after SCI (Figure 8a and c) and quantified the apoptosis rate (Figure 8b and d). By observing the fluorescence morphology, we found significantly more Tunel cells in the SCT group compared to that in the Sham group (Figure 8a and c), and the apoptosis rate was significantly higher (Figure 8b). There were more Tunel cells in the Netrin-1-si group compared to that in the NC group (Figure 8a), and the apoptosis rate was significantly higher (Figure 8b). The Netrin-1-ORF group had fewer Tunel cells (Figure 8c) and showed a significant decrease in the apoptosis rate (Figure 8d). These results indicate that high Netrin-1 expression inhibited apoptosis on day 7 during the subacute phase of SCI. Finally, we used BBB to assess the recovery of motor function in rats after SCI. The Sham group started showing a normal BBB score on day 1 after SCI, with a score of 21. From day 7 after SCI induction, the rats in the Netrin-1-ORF group started showing significantly better hind limb motor function compared to the rats in the NC group. The rats in the Netrin-1-si group showed significantly slower recovery of the motor function of the hind limbs compared to the rats in the NC group (Figure 9). These results suggested that increasing the expression of Netrin-1 contributes to the long-term motor function recovery in rats.

### 3.5 Changes in the expression of DCC after altering Netrin-1 expression in spinal cord tissue

**Figure 10:**
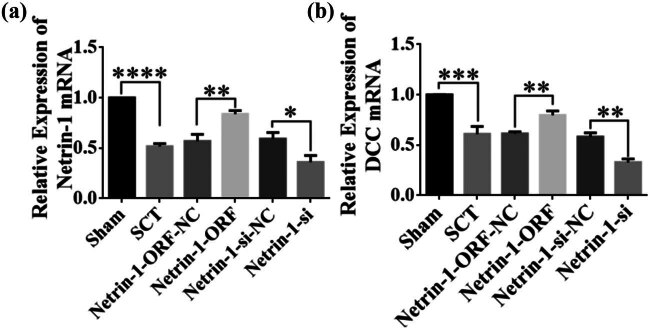
Relationship between Netrin-1 overexpression and low expression after SCI in spinal cord tissue and DCC expression. (a) Horizontal coordinates: Sham group, SCT group, Netrin-1-ORF-NC group, Netrin-1-ORF group, Netrin-1-si-NC group, and Netrin-1-si group; vertical coordinates indicate the relative mRNA expression of Netrin-1. *p < 0.05, **p < 0.01, and ****p < 0.0001, statistically significant. (b) Horizontal coordinates: Sham group, SCT group, Netrin-1-ORF-NC group, Netrin-1-ORF group, Netrin-1-si-NC group, and Netrin-1-si group; vertical coordinates indicate the relative mRNA expression of DCC expression. **p < 0.01 and ***p < 0.001, statistically significant.

The constructed Netrin-1 overexpression and Netrin-1 low-expression lentiviruses were injected into the spinal cord at the upper and lower ends of the site of SCI. PCR was performed 7 days after SCI to determine the infection efficiency and the expression of DCC. The results showed that the expression of Netrin-1 and DCC was significantly lower in the SCT group than in the Sham group (Figure 10a and b). In the SCI group compared to the control group, the relative expression of Netrin-1 mRNA in spinal cord tissue increased after Netrin-1 was overexpressed and decreased in primary spinal cord neuronal cells after its expression decreased (Figure 10a). These findings indicated that the expression of Netrin-1 increased and decreased in the spinal cord of rats in the respective treatment groups. The expression of the DCC mRNA in spinal cord tissue increased after Netrin-1 was overexpressed and decreased after its expression decreased, compared to the expression of DCC mRNA in the control group (Figure 10b), which indicated that the expression of the DCC mRNA increased or decreased after the overexpression or low expression of Netrin-1. These results suggested that Netrin-1 is positively correlated with the expression of its receptor DCC. Therefore, further studies on the role of DCC in damaged spinal cord are needed.

### 3.6 Expression of the DCC lentiviruses in the spinal cord after SCI

**Figure 11:**
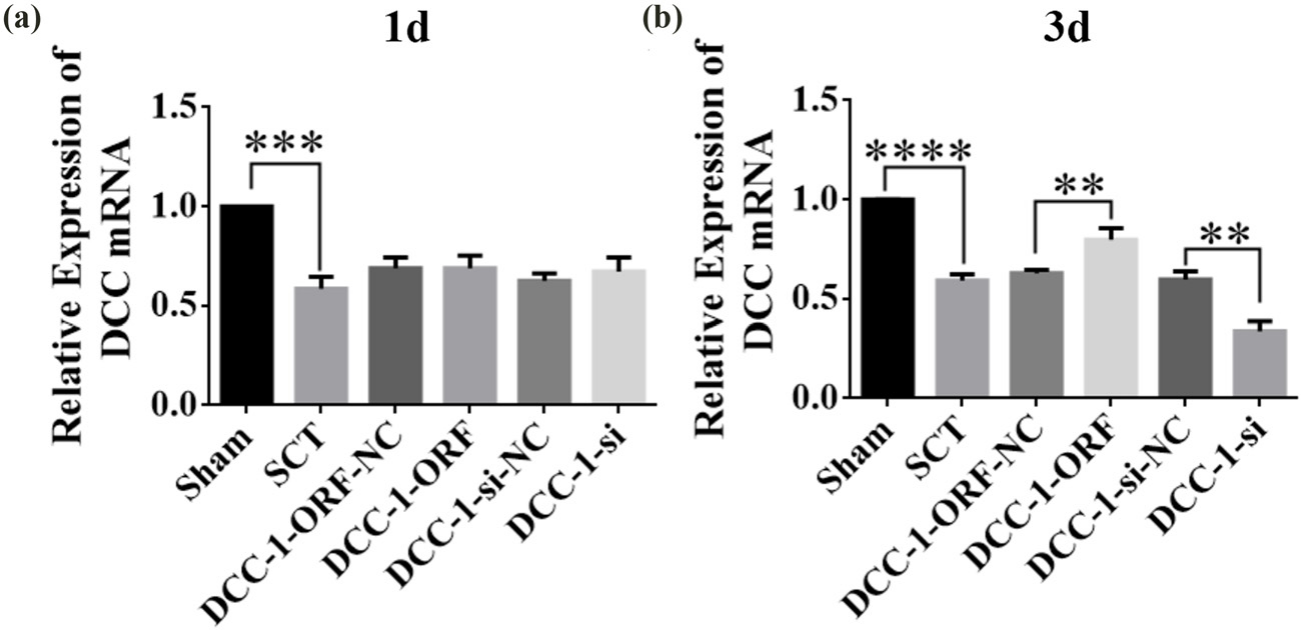
Expression of DCC overexpression/low expression lentivirus at 1 and 3 days after SCI. (a) 1 day after SCI, horizontal coordinates: Sham group, SCT group, DCC-ORF-NC group, DCC-ORF group, DCC-si-NC group, and DCC-si group; vertical coordinates indicate the relative mRNA expression of DCC. (b) 3 days after SCI, horizontal coordinates: Sham group, SCT group, DCC-ORF-NC group, DCC-ORF group, DCC DCC-NC group, and DCC-si group; vertical coordinates indicate the relative mRNA expression of DCC. Results are expressed as mean ± SD. Ordinary one-way ANOVA and LSD tests were performed: **p < 0.01, ***p < 0.001, and ****p < 0.0001.

The DCC overexpression/low-expression lentiviruses were injected into the spinal cord immediately after spinal cord transection at the upper and lower ends of the transection site. The DCC levels were assessed by PCR 2 days after SCI. The expression of DCC was significantly lower compared to that in the Sham group (Figure 11a). The expression of DCC in the DCC overexpression group was higher than that in the control group, but the difference was not significant. The expression of DCC in the DCC low-expression group was lower than that in the control group, but the difference was not significant (Figure 11a). Three days after SCI, the results of the PCR assay showed that the expression of DCC was significantly lower than that in the Sham group (Figure 11b). The expression of DCC was significantly higher in the DCC overexpression group compared to that in the control group. The expression of DCC was significantly lower in the DCC low-expression group compared to that in the control group (Figure 11b). Therefore, these results indicated that DCC overexpression/low-expression lentiviruses were not fully expressed in spinal cord tissues 1 day after SCI. Their expression started 3 days after SCI. We waited till the expression of DCC started in spinal cord tissues before testing its role after SCI; therefore, the function of DCC was tested in the subacute and chronic phases of SCI.

### 3.7 DCC alters the morphology and viability of neurons in spinal cord tissue and rat motor function after SCI

**Figure 12:**
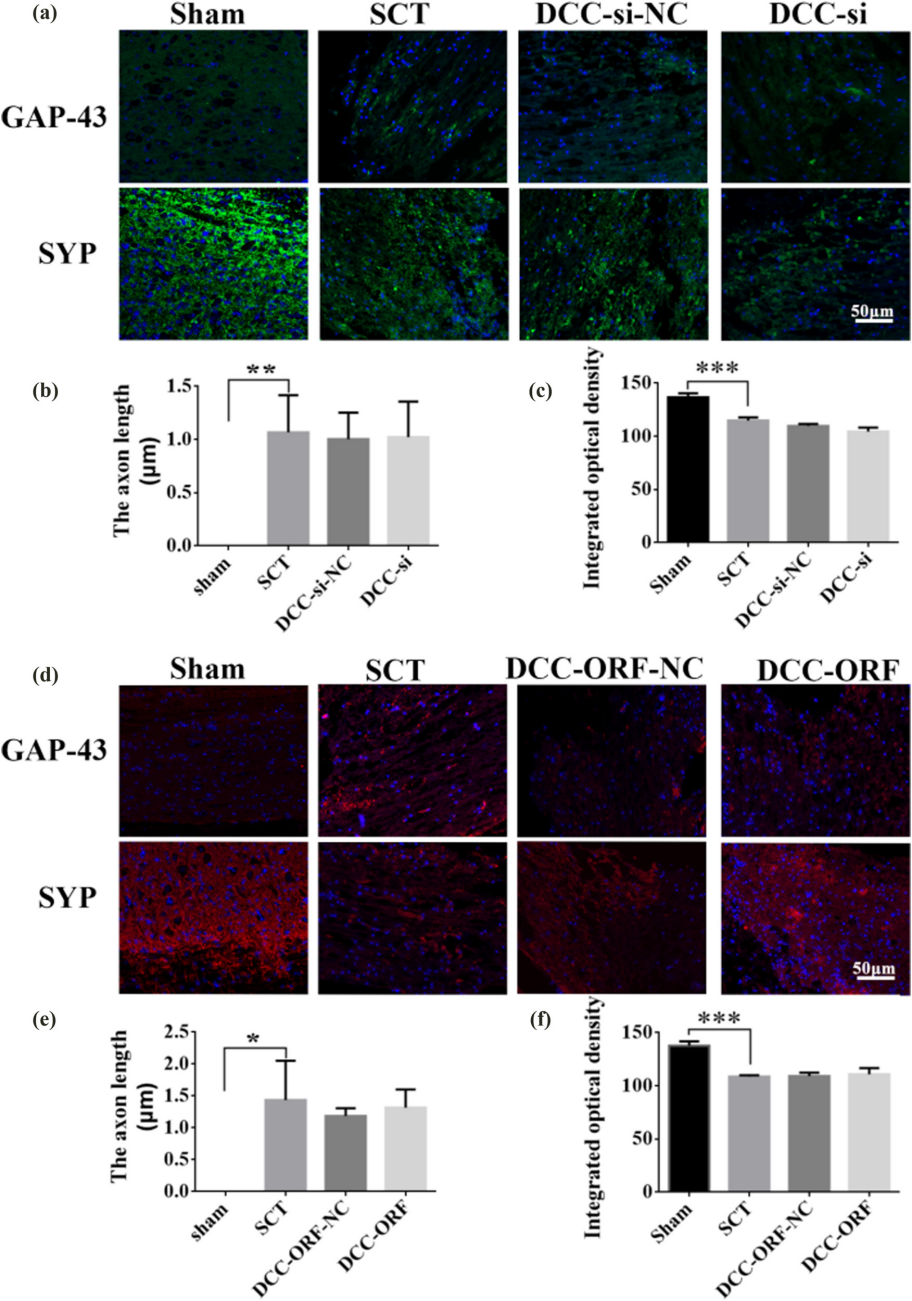
Histological analysis of DCC expression increased/decreased after 1 day post-SCI. (a) Immunohistochemical staining was used to determine GAP43 (neurospecific protein associated with synaptic development and neuronal regeneration, green) and SYP (synaptophysin, green) expression. DAPI: nuclear marker (blue), bar = 50 µm. (b) and (c) Quantitative statistics of GAP43-positive nascent axons and fluorescence intensity of SYP-positive in Sham, SCT, DCC-si-NC, and DCC-si groups. *n* = 6. (d) Immunohistochemical staining was used to determine GAP43 (red) and SYP (red) expression. DAPI (blue), bar = 50 µm. (e) and (f) Quantitative statistics of GAP43-positive nascent axons and fluorescence intensity of SYP-positive in Sham, SCT, DCC-ORF-NC, and DCC-ORF groups. *n* = 6. Results are expressed as mean ± SD. Ordinary one-way ANOVA and LSD tests were performed: **p < 0.01, ***p < 0.001, and ****p < 0.0001.

**Figure 13:**
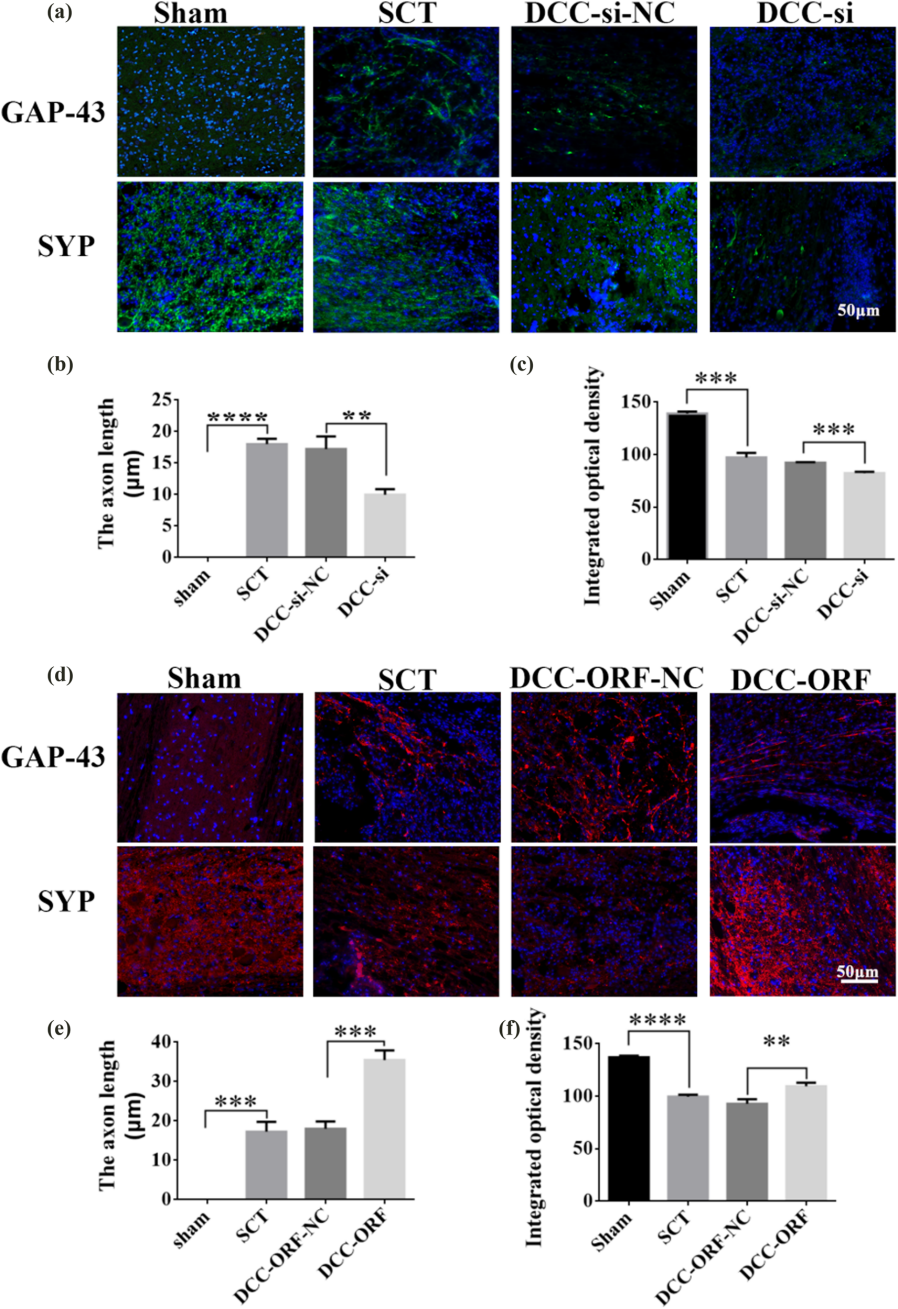
Histological analysis of DCC expression increased/decreased after 7 days post-SCI. (a) Immunohistochemical staining was used to determine GAP43 (neurospecific protein associated with synaptic development and neuronal regeneration, green) and SYP (synaptophysin, green) expression. DAPI: nuclear marker (blue), bar = 50 µm. (b) and (c) Quantitative statistics of GAP43-positive nascent axons and fluorescence intensity of SYP-positive in Sham, SCT, DCC-si-NC, and DCC-si groups. *n* = 6, (d) Immunohistochemical staining was used to determine GAP43 (red) and SYP (red) expression. DAPI (blue), bar = 50 µm. (e) and (f) Quantitative statistics of GAP43-positive nascent axons and fluorescence intensity of SYP-positive in Sham, SCT, DCC-ORF-NC, and DCC-ORF groups. *n* = 6. Results are expressed as mean ± SD. Ordinary one-way ANOVA and LSD tests were performed: **p < 0.01, ***p < 0.001, and ****p < 0.0001.

**Figure 14:**
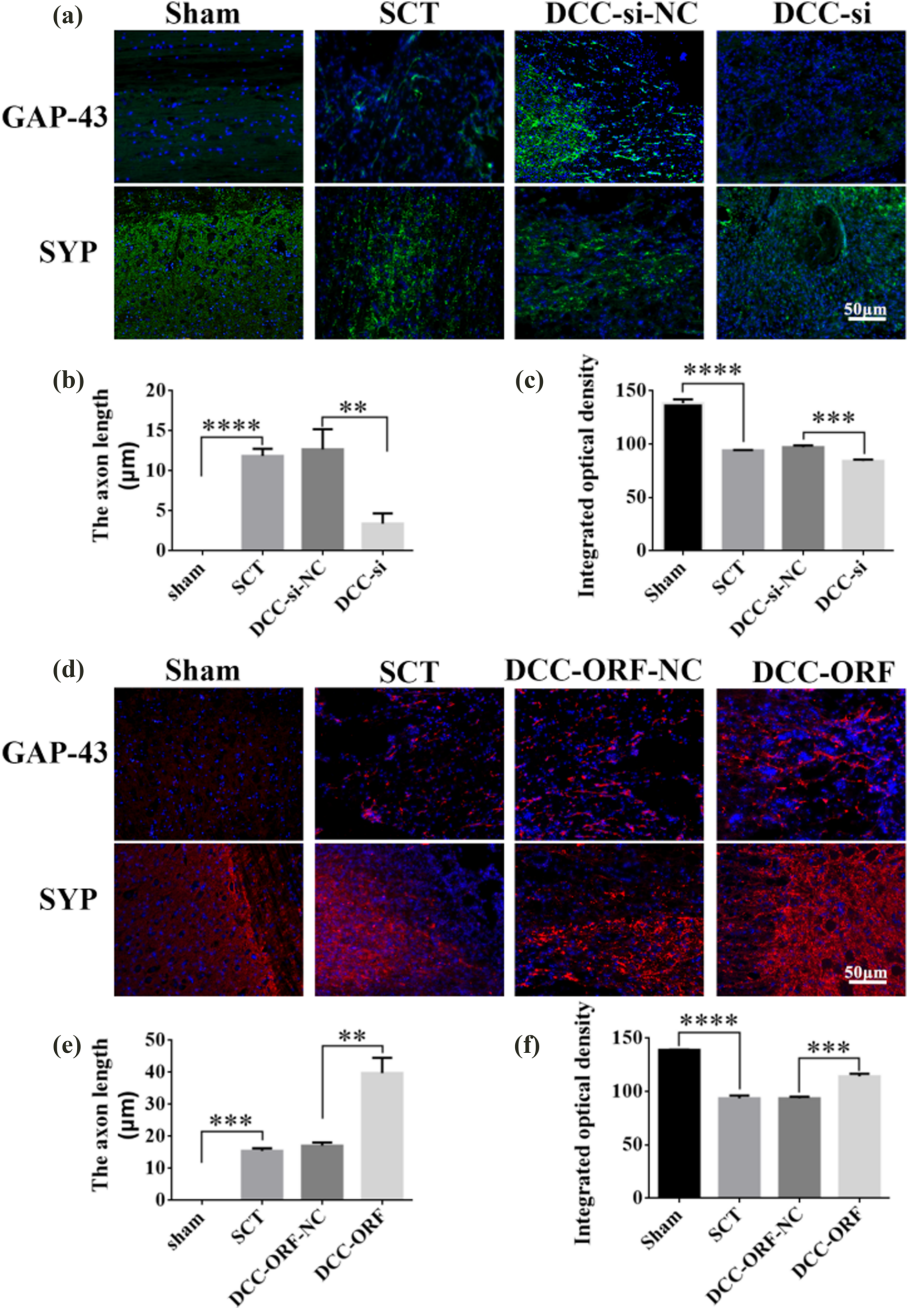
Histological analysis of DCC expression increased/decreased after 14 days post-SCI. (a) Immunohistochemical staining was used to determine GAP43 (neurospecific protein associated with synaptic development and neuronal regeneration, green) and SYP (synaptophysin, green) expression. DAPI: nuclear marker (blue), bar = 50 µm. (b) and (c) Quantitative statistics of GAP43-positive nascent axons and fluorescence intensity of SYP-positive in Sham, SCT, DCC-si-NC, and DCC-si groups. *n* = 6. (d) Immunohistochemical staining was used to determine GAP43 (red) and SYP (red) expression. DAPI (blue), bar = 50 µm. (e) and (f) Quantitative statistics of GAP43-positive nascent axons and fluorescence intensity of SYP-positive in Sham, SCT, DCC-ORF-NC, and DCC-ORF groups. *n* = 6. Results are expressed as mean ± SD. Ordinary one-way ANOVA and LSD tests were performed: **p < 0.01, ***p < 0.001, and ****p < 0.0001.

**Figure 15:**
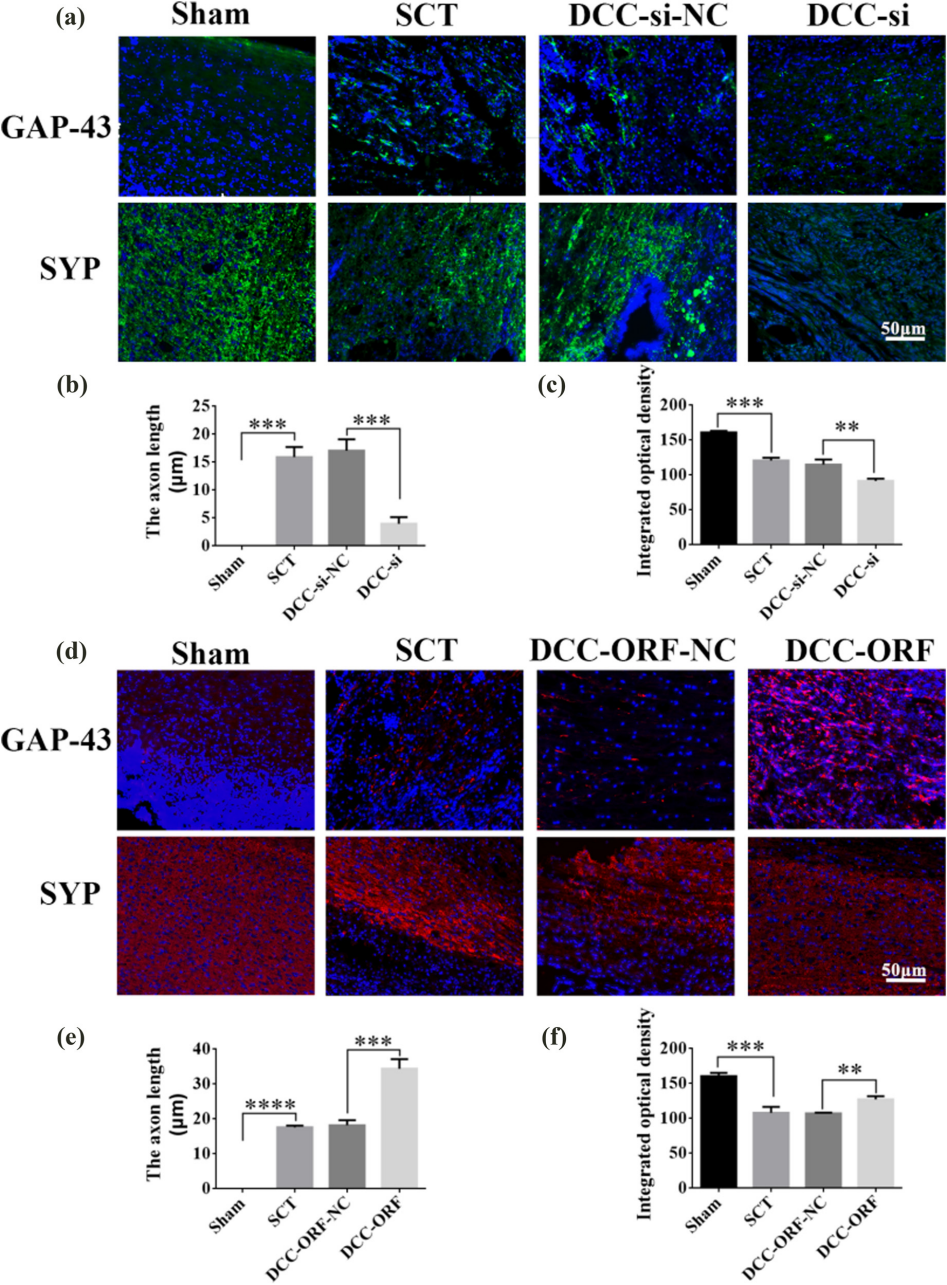
Histological analysis of DCC expression increased/decreased after 28 days post-SCI. (a) Immunohistochemical staining was used to determine GAP43 (neurospecific protein associated with synaptic development and neuronal regeneration, green) and SYP (synaptophysin, green) expression. DAPI: nuclear marker (blue), bar = 50 µm. (b) and (c) Quantitative statistics of GAP43-positive nascent axons and fluorescence intensity of SYP-positive in Sham, SCT, DCC-si-NC, and DCC-si groups. *n* = 6. (d) Immunohistochemical staining was used to determine GAP43 (red) and SYP (red) expression. DAPI (blue), bar = 50 µm. (e) and (f) Quantitative statistics of GAP43-positive nascent axons and fluorescence intensity of SYP-positive in Sham, SCT, DCC-ORF-NC, and DCC-ORF groups. *n* = 6. Results are expressed as mean ± SD. Ordinary one-way ANOVA and LSD tests were performed: **p < 0.01, ***p < 0.001, and ****p < 0.0001.

**Figure 16:**
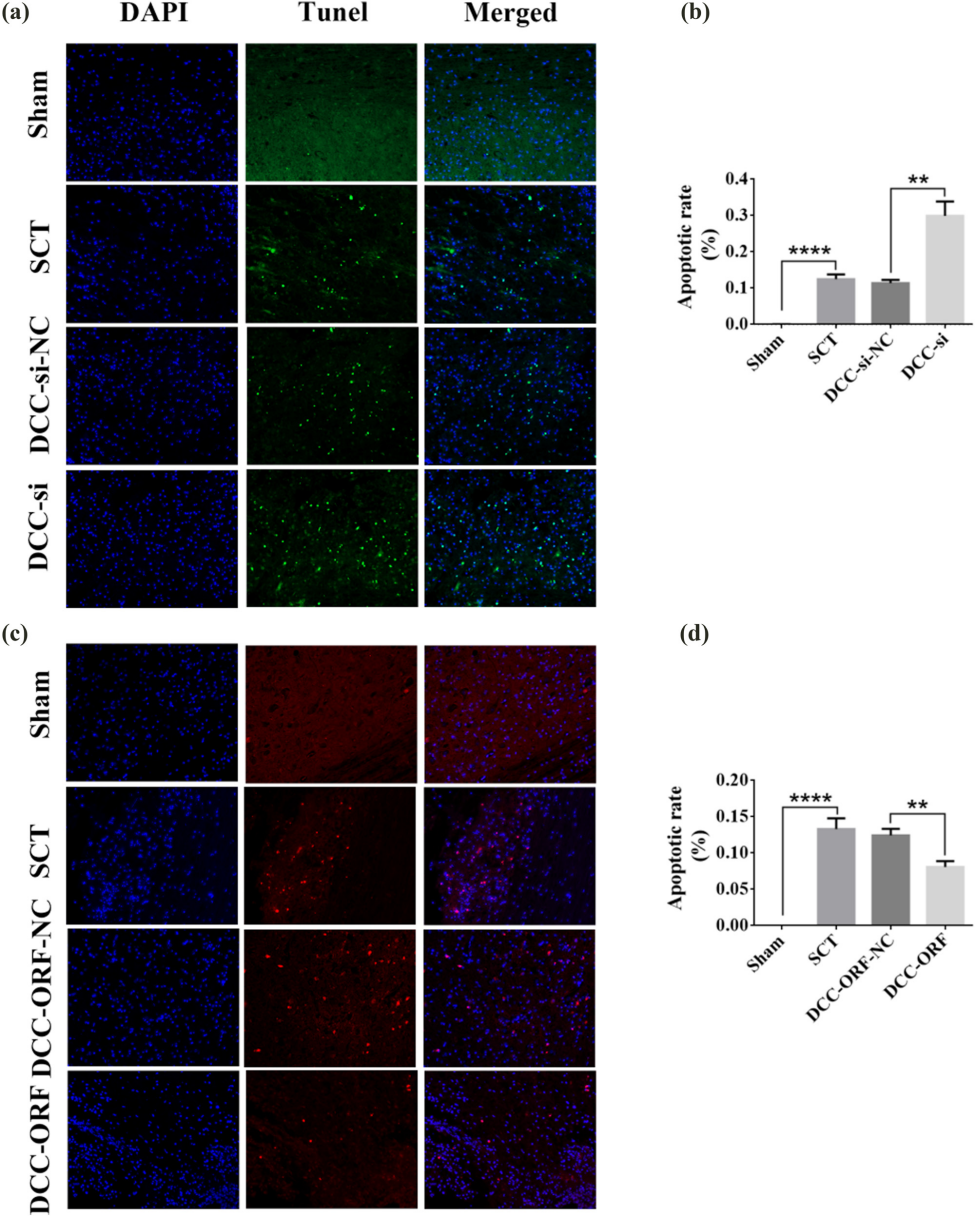
Tunel analysis of DCC expression increase/decrease after 7 days post-SCI. (a) DAPI (blue) indicates the nucleus, and Tunel (green) indicates apoptosis. From left to right: DAPI, Tunel, TUNEL/DAPI Merged (fusion plot); from top to bottom, Sham group, SCT group, DCC-si-NC group, and DCC-si group under SCT conditions; images are 200×, bar = 50 μm. (b) For Sham, SCT, DCC-si-NC, and DCC-si groups with quantitative statistics of apoptosis rate. *n* = 6. (c) DAPI (blue) indicates nuclei, and Tunel (red) indicates apoptosis. From left to right, DAPI, Tunel, and TUNEL/DAPI Merged (fusion plot); from top to bottom, Sham group, SCT group, DCC-ORF-NC group under SCT conditions, and DCC-ORF group, respectively; images are 200×, bar = 50 μm. (d) Quantification of apoptosis rates for Sham, SCT, DCC-ORF-NC, and DCC-ORF groups were statistically analyzed. *n* = 6. Results are expressed as mean ± SD. Ordinary one-way ANOVA and LSD tests were performed: **p < 0.01 and ****p < 0.0001.

**Figure 17:**
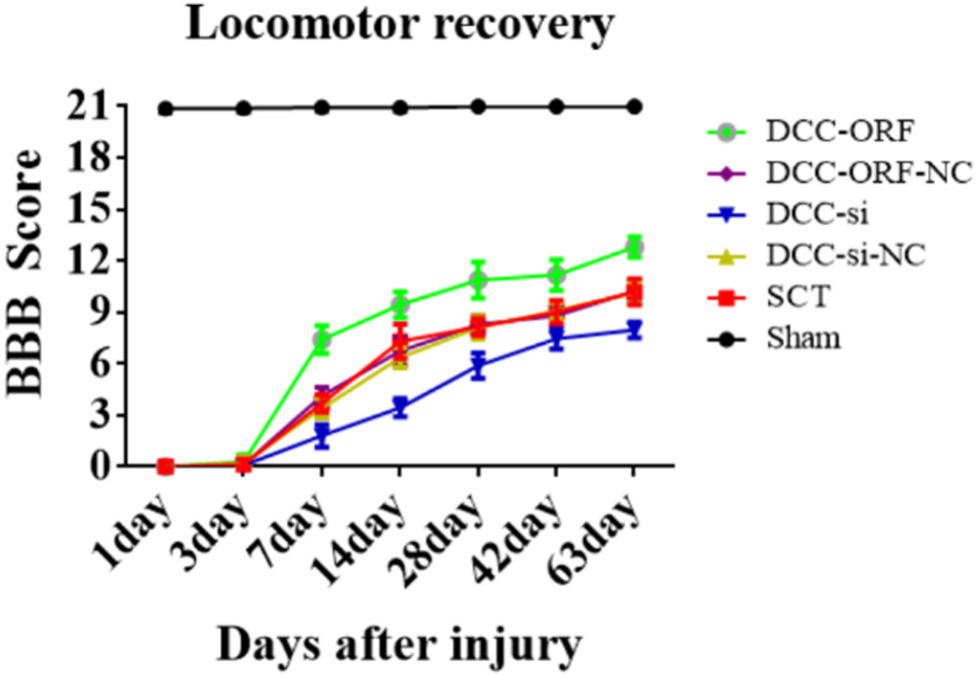
Behavioral analysis after increase/decrease of DCC expression. The abscissa represents 1, 3, 7, 14, 28, 42, and 63 days, and the ordinate represents the BBB score.

On days 1, 7, 14, and 28 after SCI, we examined the morphological changes in GAP43 and SYP cells (Figure 12a and d). We also quantified the length of nascent neuronal axons and the fluorescence intensity of positive cells (Figure 12b, c, e, and f). The axons started becoming neoplastic 1 day after SCT, but an increase or decrease in the expression of DCC did not significantly affect the treatment of axonal regeneration after SCI. Higher DCC expression facilitated axonal regeneration in the subacute and chronic phases of SCI (Figures 13–17). As the role of DCC in promoting axonal regeneration was detected starting 7 days after SCI, we examined the morphological changes in Tunel cells 7 days after SCI (Figure 16a and c) and quantified the apoptosis rate (Figure 16b and d). Using fluorescence techniques, we found significantly more Tunel cells in the SCT group compared to that in the Sham group (Figure 16a and c) and a significant increase in the apoptosis rate (Figure 16b). More Tunel cells were recorded in the DCC-si group compared to that in the NC group (Figure 16a), and a significant increase in the apoptosis rate (Figure 16b). However, the number of Tunel cells was lower in the DCC-ORF group (Figure 16c), and the apoptosis rate was significantly lower (Figure 16d). These results indicated that increasing the expression of DCC inhibited apoptosis on day 7 in the subacute phase of SCI. Finally, we used BBB to assess the recovery of motor function on days 1, 3, 7, 14, and 28 after SCI in rats. The Sham group achieved a normal BBB score on day 1 after SCI, with a score of 21. Starting from day 7 after SCI, the rats in the DCC-ORF group showed significantly better motor function of the hind limbs compared to those in the NC group. The rats in the DCC-si group had significantly slower recovery of motor function of the hind limbs compared to those in the NC group (Figure 17). These results indicated that increasing the expression of DCC can contribute to the recovery of long-term motor functions in rats.

### 3.8 Changes in the expression of Netrin-1 and DCC affect motor function after SCI

**Figure 18:**
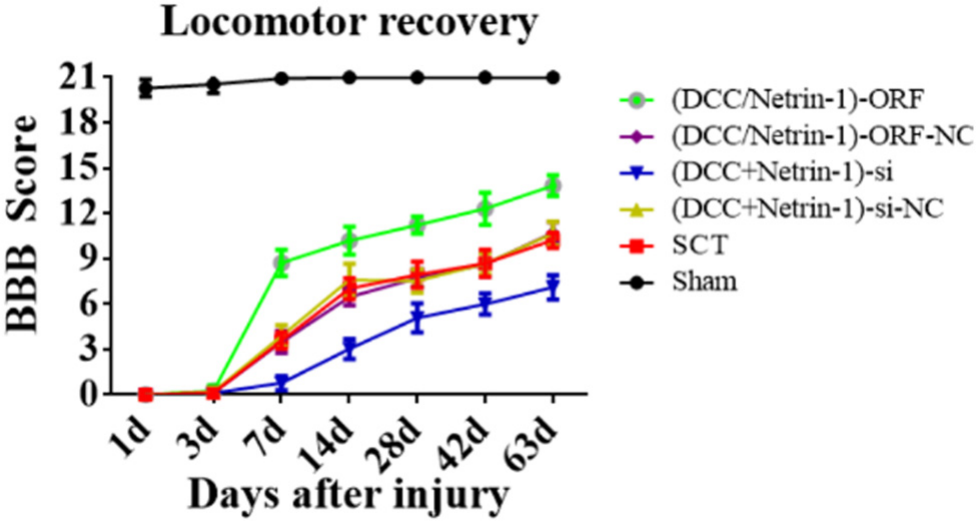
Behavioral analysis after co-overexpression/low expression of Netrin-1 and DCC. The abscissa represents 1, 3, 7, 14, 28, 42, and 63 days, and the ordinate represents the BBB score.

After the co-overexpression/low expression of Netrin-1 and DCC in rats with SCI, we used BBB to assess motor function recovery on days 1, 3, 7, 14, 28, 42, and 63. The BBB score in the Sham group showed a normal value of 21 3 days after SCI. From day 7 after SCI, rats in the DCC/Netrin-1 ORF group started showing significant improvements in the motor function of the hind limbs compared to the rats of the NC group; however, the rats in the DCC/Netrin-1-si group showed significantly slower recovery of motor function in the hind limbs compared to the rats in the NC group (Figure 18). These results suggested that Netrin-1 contributes to long-term motor function recovery in rats after binding to its receptor DCC. The previous results indicated that Netrin-1 and its receptor DCC promote axonal regeneration and attenuate apoptosis in the subacute and chronic phases of SCI in rats, and the combination of the two also significantly promotes the recovery of motor function. However, the mechanism underlying their effects needs to be investigated.

### 3.9 PCR validation of the regulatory relationship between the factors related to the NgR1 pathway and effects of changes in Netrin-1 and DCC expression on axonal regeneration-related factors GAP43 and SYP

**Figure 19:**
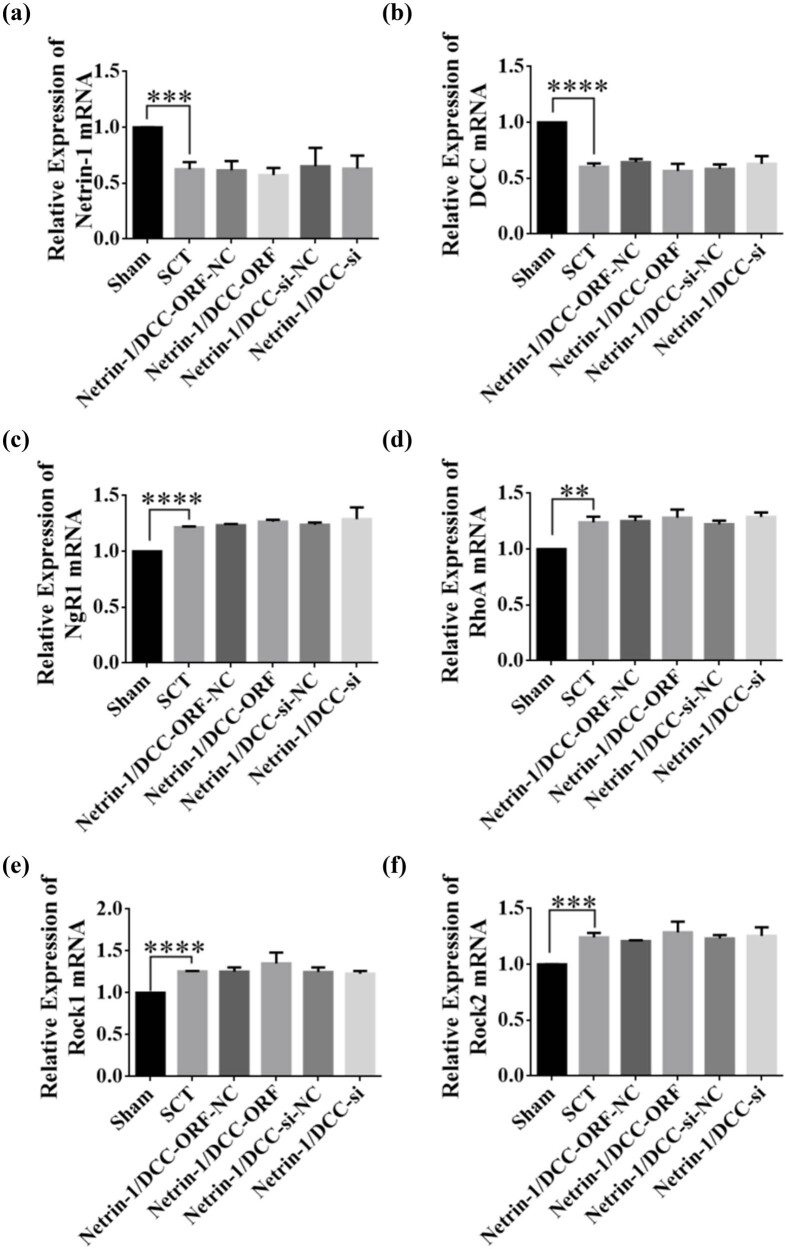
Change in mRNA expression of NgR1 pathway factor after co-overexpression/low expression of Netrin-1 and DCC at 1 day after SCI. (a–f) Horizontal coordinates: Sham group, SCT group, Netrin-1 and DCC co-overexpression control group, Netrin-1 and DCC co-overexpression group, Netrin-1 and DCC co-low expression control group, and Netrin-1 and DCC co-low expression group 1 day after SCI; vertical coordinates: Netrin-1, DCC, NgR1, RhoA, Rock1, and Rock2 relative mRNA expressions. **p < 0.01, ***p < 0.001, and****p < 0.0001, statistically significant.

Immediately after SCI, the expression of Netrin-1 and DCC decreased significantly, and the expression of NgR1, RhoA, Rock1, and Rock2 mRNAs increased significantly in the SCT group compared to their respective levels in the Sham group (Figures 19a and b). DCC co-low-expression lentiviruses were injected into the upper and lower parts of the site of SCI immediately after the injury, and the results of RT-PCR assays 1 day after SCI showed that the viruses did not infect successfully (Figure 19a and b). The expression of the NgR1, RhoA, Rock1, and Rock2 mRNAs in the Netrin-1 and DCC co-low-expression group did not change significantly compared to their expression in the control group. The expression of NgR1, RhoA, Rock1, and Rock2 mRNAs in the DCC co-overexpression group was not significantly different compared to that in the control group (Figure 19c–f). These results indicated that 1 day after SCI, the Netrin-1 and DCC co-overexpressing/low-expressing lentiviruses did not infect the rats, and thus, no relationship with the factors related to the NgR1 pathway was detected.

**Figure 20:**
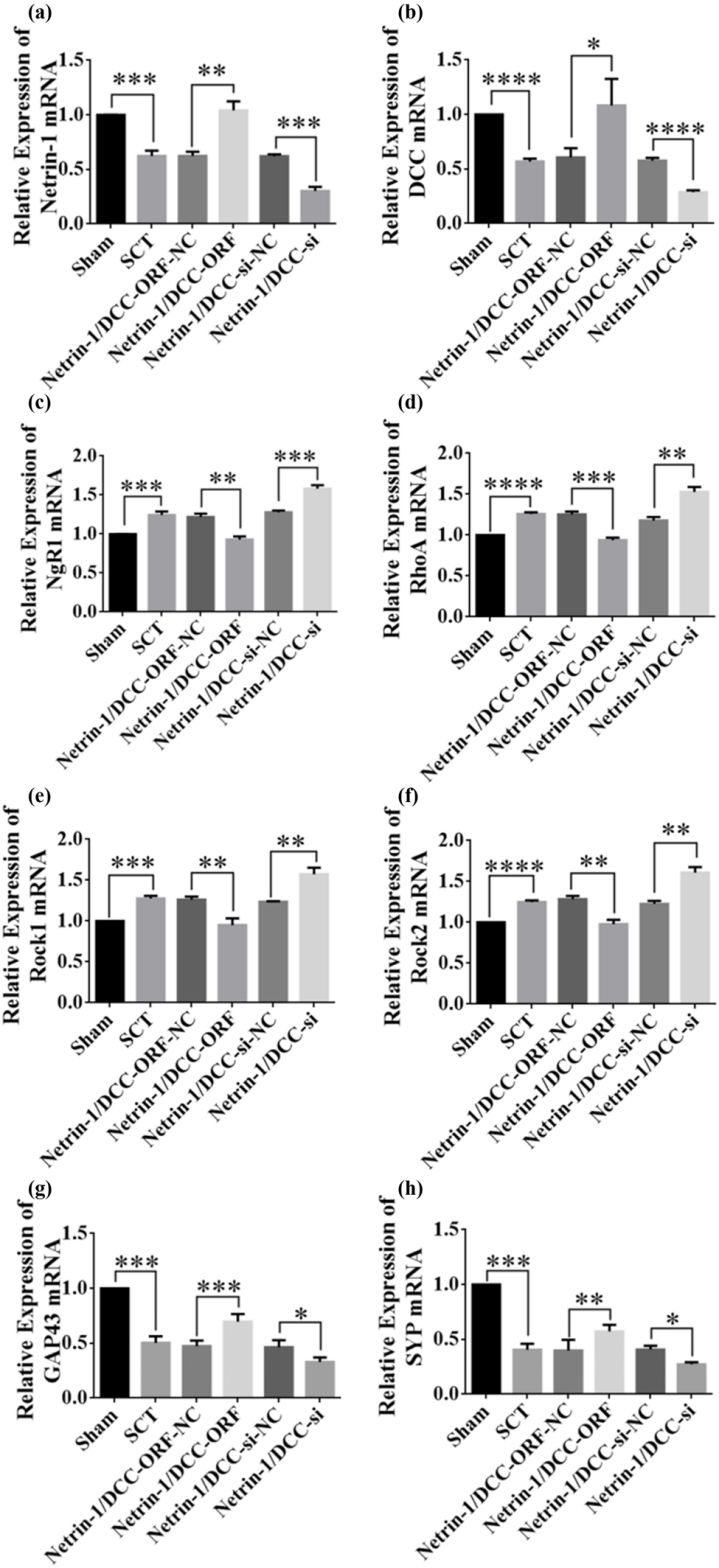
Relative expression of mRNAs in NgR1 pathway factors and GAP43 and SYP after co-overexpression/low expression of Netrin-1 and DCC at 7 days after SCI. (a)–(h) Horizontal coordinates: Sham group, SCT group, Netrin-1 and DCC co-overexpression control group, Netrin-1 and DCC co-overexpression group, Netrin-1 and DCC co-low expression control group, and Netrin-1 and DCC co-low expression group 7 days after SCI; vertical coordinates: Netrin-1, DCC, NgR1, RhoA, Rock1, Rock2, GAP43, and SYP relative mRNA expressions. **p < 0.01, ***p < 0.001, and ****p < 0.0001, statistically significant.

**Figure 21:**
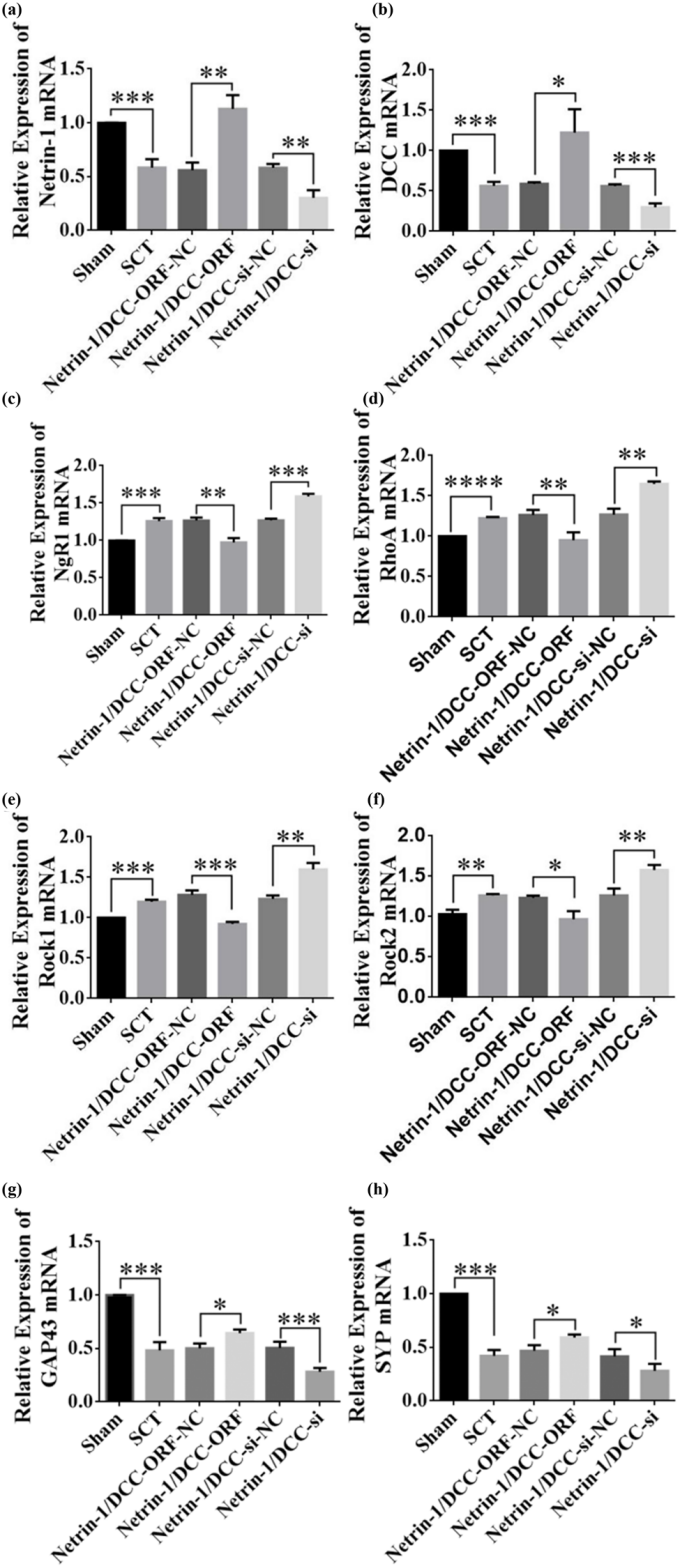
Relative expression of mRNAs in NgR1 pathway factors and GAP43 and SYP after co-overexpression/low expression of Netrin-1 and DCC at 14 days after SCI. (a)–(h) Horizontal coordinates: Sham group, SCT group, Netrin-1 and DCC co-overexpression control group, Netrin-1 and DCC co-overexpression group, Netrin-1 and DCC co-low expression control group, and Netrin-1 and DCC co-low expression group 14 day after SCI; vertical coordinates: Netrin-1, DCC, NgR1, RhoA, Rock1, Rock2, GAP43, and SYP relative mRNA expressions. **p < 0.01, ***p < 0.001, and ****p < 0.0001, statistically significant.

**Figure 22:**
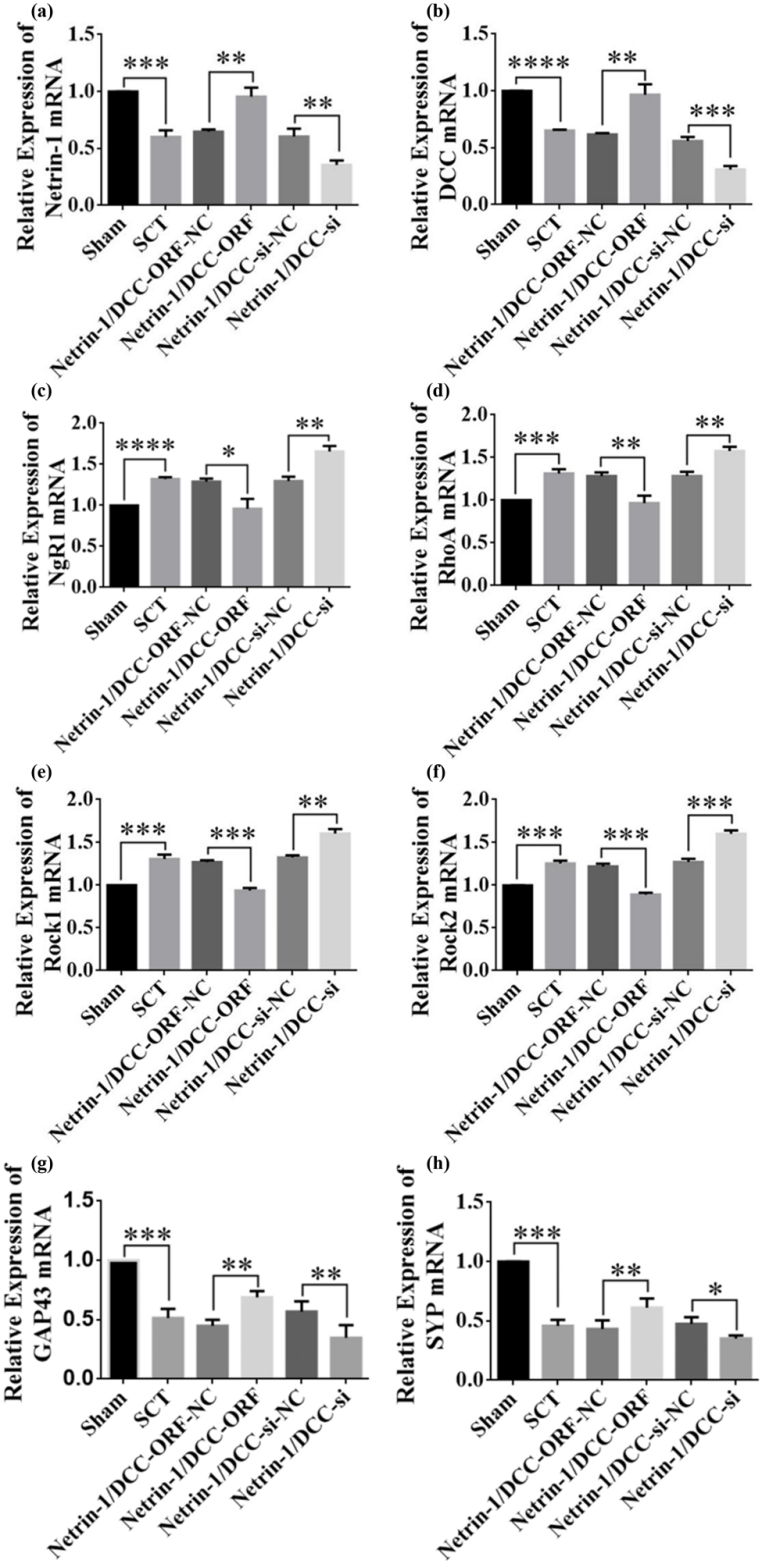
Relative expression of mRNAs in NgR1 pathway factors and GAP43 and SYP after co-overexpression/low expression of Netrin-1 and DCC at 28 days after SCI. (a)–(h) Horizontal coordinates: Sham group, SCT group, Netrin-1 and DCC co-overexpression control group, Netrin-1 and DCC co-overexpression group, Netrin-1 and DCC co-low expression control group, and Netrin-1 and DCC co-low expression group 28 day after SCI; vertical coordinates: Netrin-1, DCC, NgR1, RhoA, Rock1, Rock2, GAP43, and SYP relative mRNA expressions. **p < 0.01, ***p < 0.001, and ****p < 0.0001, statistically significant.

The results of RT-PCR assays from days 7 to 28 after SCI showed successful viral infection (Figures 20 and 22a and b). The expression of the NgR1, RhoA, Rock1, and Rock2 mRNAs was higher in the SCT group compared to that in the Sham group. In contrast, the expression of the NgR1, RhoA, Rock1, and Rock2 mRNAs was significantly lower in the Netrin-1 and DCC co-overexpression group compared to that in the control group. The Netrin-1 and DCC co-low-expression group showed higher expression of NgR1, RhoA, Rock1, and Rock2 mRNAs than the control group. This finding indicated that Netrin-1 and DCC levels were negatively associated with factors related to the NgR1 pathway after co-expression/low expression on days 7–28 after SCI. Netrin-1 and DCC may act as promoters of axon regeneration by inhibiting the downstream pathway factors NgR1, RhoA, Rock1, and Rock2. The effect of Netrin-1 and DCC on axon regeneration after co-low expression and overexpression can be illustrated by detecting GAP43 and SYP. The expression of GAP43 and SYP proteins was lower after SCT compared to their levels in the Sham group. The expression of GAP43 and SYP proteins was higher in the Netrin-1 and DCC co-overexpression group compared to that in the Netrin-1 and DCC co-overexpression control group. The relative protein expression of GAP43 and SYP was lower in the Netrin-1 and DCC co-overexpression group compared to that in the Netrin-1 and DCC co-overexpression control group (Figures 20–22). These results showed that Netrin-1 and DCC may promote axon regeneration by inhibiting factors such as NgR1, RhoA, Rock1, and Rock2 in the downstream pathway during the subacute phase (7 days), subacute phase (14 days), and chronic phase (28 days) of SCI.

### 3.10 Regulation of the NgR1 pathway and effects of changes in Netrin-1 and DCC expression on the axonal regeneration-related factors GAP43 and SYP

**Figure 23:**
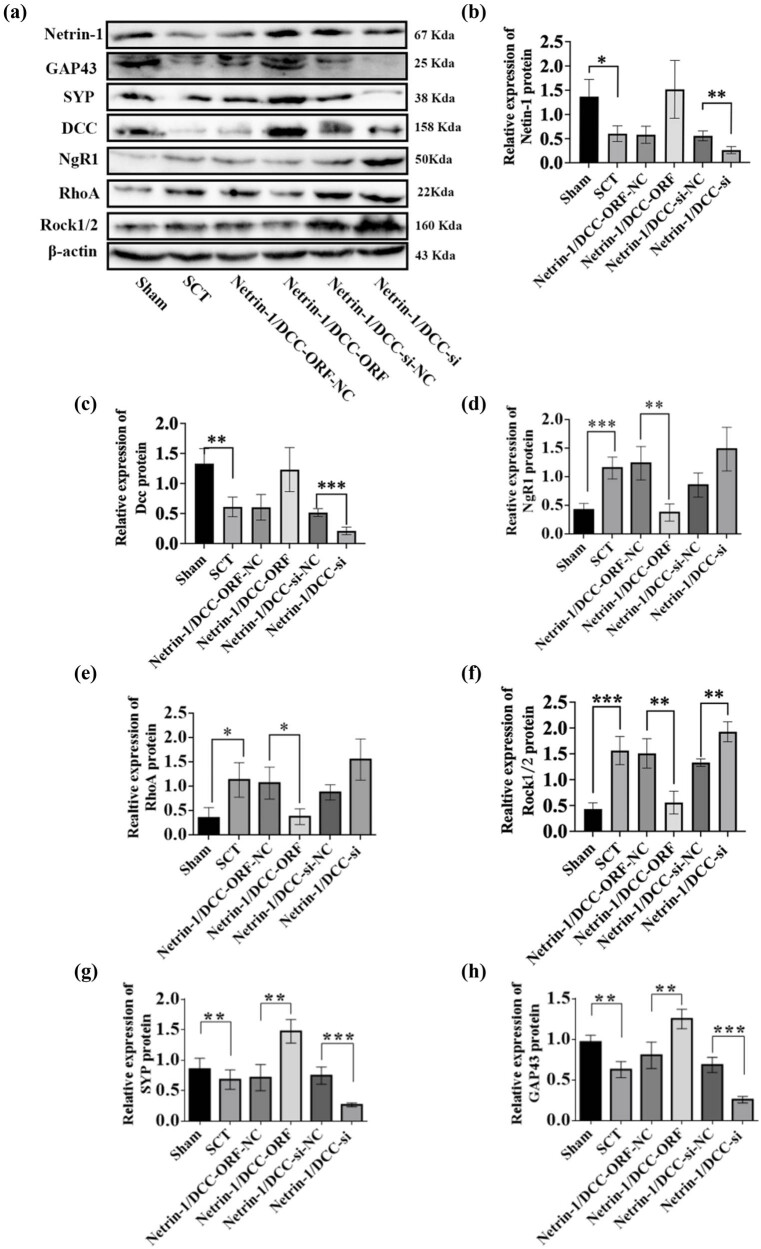
Relative expression of proteins in NgR1 pathway factors, GAP43, and SYP after Netrin-1 and DCC co-overexpression/low expression at 7 days after SCI. (a) Seven days after SCI, from left to right, the spinal cord tissues of Sham group, Netrin-1 and DCC co-overexpression control group, Netrin-1 and DCC co-overexpression group, Netrin-1 and DCC co-low expression control group, and Netrin-1 and DCC co-low expression group for Netrin-1, GAP43, SYP, DCC, NgR1, RhoA, Rock1/2, and β-actin in spinal cord tissue. (b–h) 7 days after SCI, horizontal coordinates: Sham group, Netrin-1 and DCC co-overexpression control group, Netrin-1 and DCC co-overexpression group, Netrin-1 and DCC co-low expression control group, and Netrin-1 and DCC co-low expression group; vertical coordinates: relative protein expression of Netrin-1, DCC, NgR1, RhoA, Rock1/2, GAP43, and SYP, respectively. Protein quantification was calibrated by β-actin, and the sample size for each group was *n* = 6. All two-way comparisons between multiple sample mean values were performed using a one-way ANOVA with a completely randomized design, and the variance was chi-squared using the LSD test. *p < 0.05, **p < 0.01, and ***p < 0.001, statistically significant.

**Figure 24:**
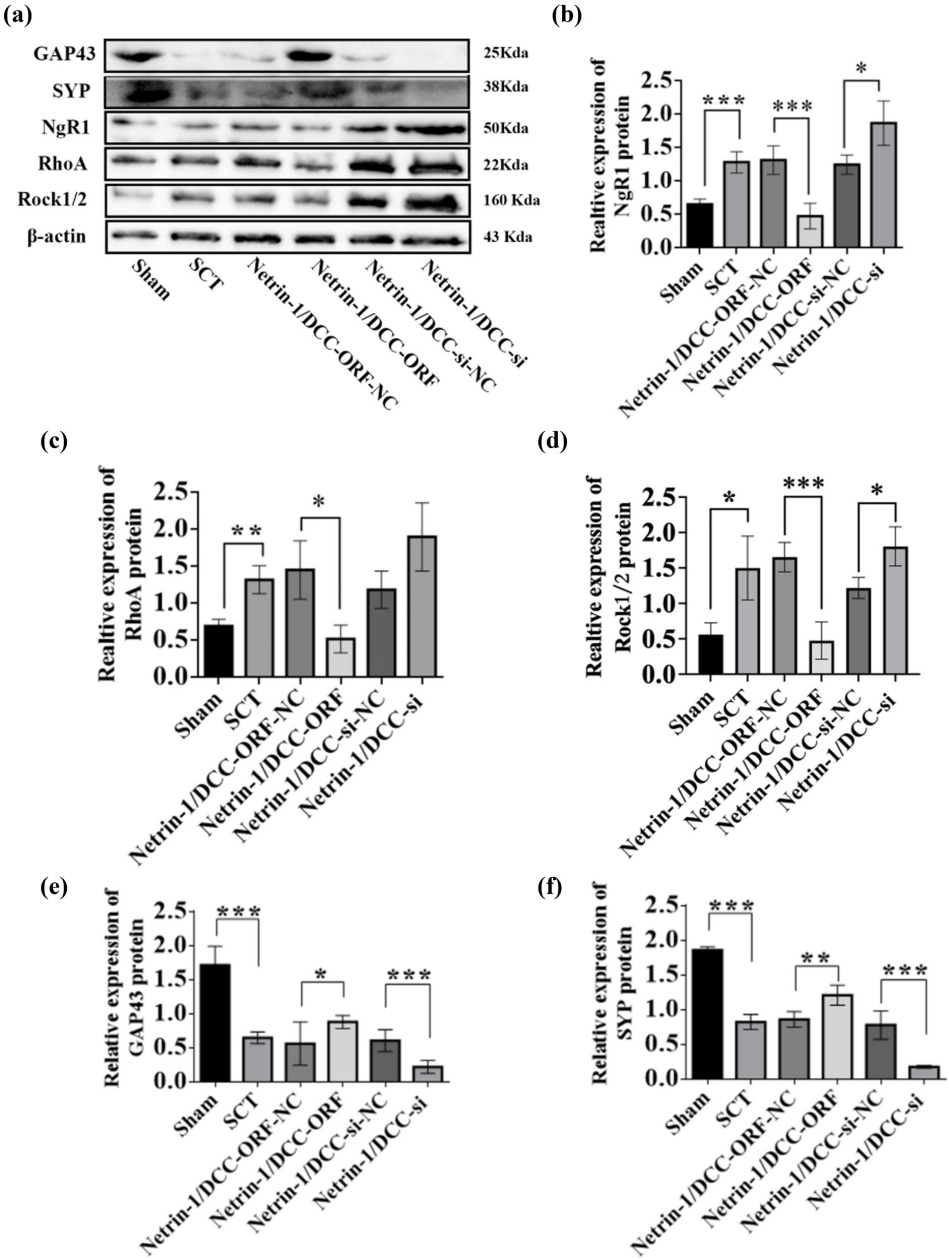
Relative expression of proteins in NgR1 pathway factors, GAP43, and SYP after Netrin-1 and DCC co-overexpression/low expression at 14 days after SCI. (a) 14 days after SCI, from left to right, the spinal cord tissues of Sham group, Netrin-1 and DCC co-overexpression control group, Netrin-1 and DCC co-overexpression group, Netrin-1 and DCC co-low expression control group, and Netrin-1 and DCC co-low expression group for Netrin-1, GAP43, SYP, DCC, NgR1, RhoA, Rock1/2, and β-actin in spinal cord tissue. (b)–(h) 14 days after SCI, horizontal coordinates: Sham group, Netrin-1 and DCC co-overexpression control group, Netrin-1 and DCC co-overexpression group, Netrin-1 and DCC co-low expression control group, and Netrin-1 and DCC co-low expression group; vertical coordinates: relative protein expression of Netrin-1, DCC, NgR1, RhoA, Rock1/2, GAP43, and SYP, respectively. Protein quantification was calibrated by β-actin, and the sample size for each group was *n* = 6. All two-way comparisons between multiple sample means were performed using a one-way ANOVA with a completely randomized design, and the variance was chi-squared using the LSD test. *p < 0.05, **p < 0.01, and ***p < 0.001, statistically significant.

**Figure 25:**
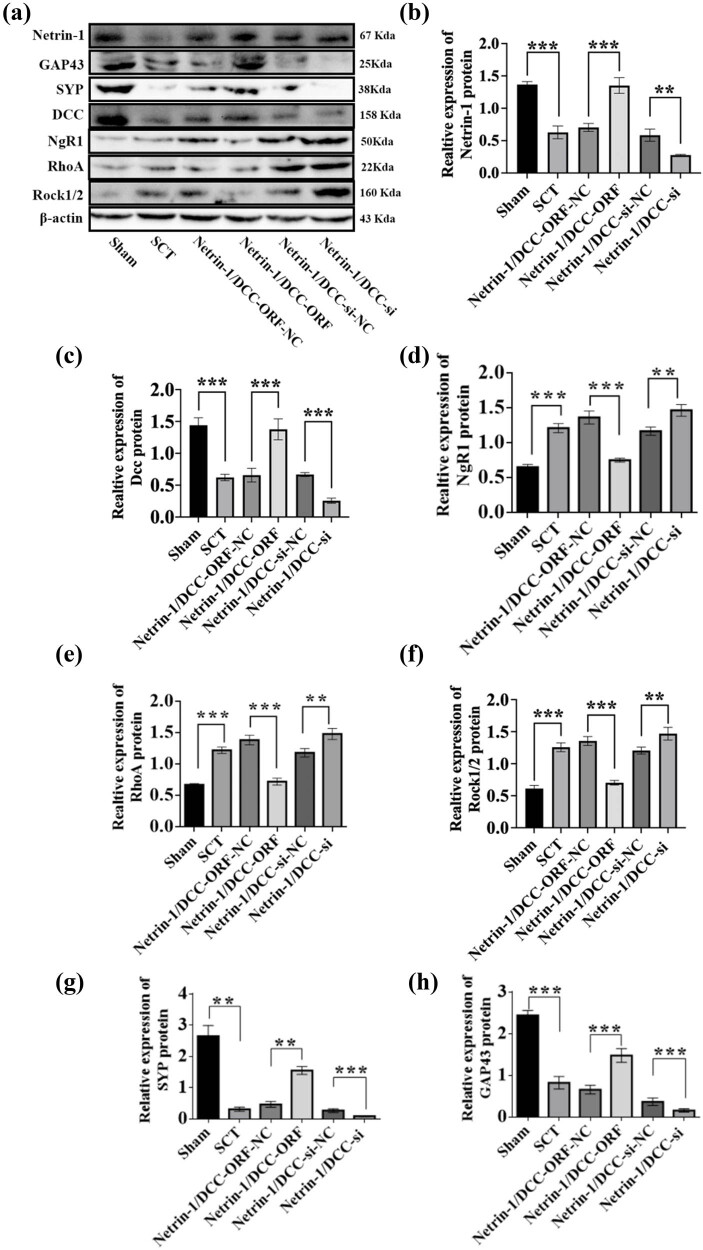
Relative expression of proteins in NgR1 pathway factors, GAP43, and SYP after Netrin-1 and DCC co-overexpression/low expression at 28 days after SCI. (a) 28 days after SCI, from left to right, the spinal cord tissues of Sham group, Netrin-1 and DCC co-overexpression control group, Netrin-1 and DCC co-overexpression group, Netrin-1 and DCC co-low expression control group, and Netrin-1 and DCC co-low expression group for Netrin-1, GAP43, SYP, DCC, NgR1, RhoA, Rock1/2, and β-actin in spinal cord tissue. (b)–(h) 28 days after SCI, horizontal coordinates: Sham group, Netrin-1 and DCC co-overexpression control group, Netrin-1 and DCC co-overexpression group, Netrin-1 and DCC co-low expression control group, and Netrin-1 and DCC co-low expression group; vertical coordinates: relative protein expression of Netrin-1, DCC, NgR1, RhoA, Rock1/2, GAP43, and SYP, respectively. Protein quantification was calibrated by β-actin, and the sample size for each group was *n* = 6. All two-way comparisons between multiple sample means were performed using a one-way ANOVA with a completely randomized design, and the variance was chi-squared using the LSD test. *p < 0.05, **p < 0.01, and ***p < 0.001, statistically significant.

We performed RT-qPCR to evaluate the negative regulatory effects of Netrin-1 and DCC after their co-overexpression/low expression on the factors in the NgR1 pathway in spinal cord tissues 7 days after the SCI model was established. Based on this, we performed WB experiments to show the changes in the expression of Netrin-1 and DCC and the expression of the proteins in the NgR1 signaling pathway after Netrin-1 and DCC co-overexpression/low expression. The results showed that the expression of the Netrin-1 and DCC proteins was significantly higher in the Netrin-1 and DCC co-overexpression group compared to that in the Netrin-1 and DCC co-overexpression control group. The expression of NgR1, RhoA, Rock1, and Rock2 proteins was lower in the Netrin-1 and DCC co-overexpression group compared to that in the Netrin-1 and DCC co-overexpression control group. The expression of Netrin-1 and DCC proteins was significantly lower in the Netrin-1 and DCC co-overexpression control group compared to that in the Netrin-1 and DCC co-low-expression control group, indicating successful viral infection. The expression of NgR1, RhoA, Rock1, and Rock2 proteins was higher in the Netrin-1 and DCC co-overexpression group compared to that in the Netrin-1 and DCC co-overexpression control group. This indicated that the spinal cord tissue was negatively associated with the factors related to the NgR1 pathway after the co-overexpression/low expression of Netrin-1 and DCC after SCI; these results were similar to those of the PCR assays. The effects of Netrin-1 and DCC on axon regeneration after co-overexpression and overexpression were evaluated by determining the levels of GAP43 and SYP proteins. The expression of GAP43 and SYP proteins decreased after SCT compared to their expression in the Sham group. The expression of GAP43 and SYP proteins was higher in the Netrin-1 and DCC co-overexpression group compared to that in the Netrin-1 and DCC co-overexpression control group. Compared to the Netrin-1 and DCC co-low expression control group, the relative protein expression of both Netrins (Figures 23–25). These results indicated that Netrin-1 and DCC may promote axon regeneration by inhibiting the downstream pathway factors NgR1, RhoA, Rock1, and Rock2 during the subacute and chronic phases of SCI.

## 4 Discussion

In this study, we investigated the function and mechanism of binding of Netrin-1 to its receptor DCC by constructing a rat spinal cord total transection model. Two main modes were used to induce SCI in rats, including complete transection and blunt weight contusion [33,34,35,36]. The complete transection model is the most commonly used method for studying SCI and is more suitable for studying regeneration, degeneration, tissue engineering strategies, and neuroplasticity, as highly reproducible and complete injury models can be established using this method [37,38]. Among them, the T10 level full transection model is the most widely used, as it can be used to reliably test and reveal the mechanism underlying axonal regeneration at the transection site [39]. To accurately assess spinal cord recovery in rats in the later stage, the spinal cord needs to undergo complete transection [40]. We evaluated the BBB score of the rats within 24 h of transection; the score was 0, which indicated that the hind limbs were completely paralyzed. Through the gross morphology after transection, we found that the nerve connections at the upper and lower ends of the site of transection were completely severed. The H & E-stained sections showed that the morphology of spinal cord neurons in the Sham group was normal, whereas most of the cells in the SCT group showed irregular morphology. These results indicated that the spinal cord neurons were injured after transection. To examine the function and mechanism associated with the binding of Netrin-1 to its receptor DCC, we constructed Netrin-1 overexpressing/low-expressing lentiviruses and DCC overexpressing/low-expressing lentiviruses and inserted them into spinal cord tissues under SCI conditions, respectively.

A study found that Netrin-1 expression is downregulated after axonal injury [41]. After injury, Netrin-1 protein can inhibit apoptosis of neurons and inflammatory response, reduce the loss of anterior horn motor neurons, promote the repair of spinal cord tissue, and ultimately, promote the recovery of motor function [42,43]. In our previous study, Netrin-1 interference and overexpression HIV recombinant plasmids were constructed. Injecting Netrin-1-overexpressing lentivirus at the site of SCI increased the expression of Netrin-1 at the injection site, which promoted the recovery of hind limb motor function and sensory function in these rats. It also increased the expression of molecules related to axonal regeneration in the injured area [44]. The results of immunofluorescence staining of GAP43 and SYP, TUNEL staining, and BBB scoring showed that the increase or decrease in the expression of Netrin-1 affected the length of axons, neuroplasticity, cell apoptosis, and the recovery of motor function after SCI. Therefore, Netrin-1 can promote SCI in rats. Additionally, DCC is a major component of the Netrin-1 receptor complex, which plays a key role in mediating growth cone elongation [45]. We found that the expression of the DCC mRNA increased or decreased with the overexpression or downregulation of Netrin-1 after the corresponding lentivirus was injected. This indicated that Netrin-1 is positively correlated with the expression of its receptor DCC. Thus, we further investigated the role of DCC in the injured spinal cord. DCC overexpression and low-expression lentiviruses were injected into rats with SCI. The results of the immunofluorescence staining of GAP43 and SYP, TUNEL staining, and BBB scoring showed that the increase or decrease in the expression of DCC affected the recovery of axonal length, neuroplasticity, cell apoptosis, and motor function after SCI. These results indicated that DCC promotes the recovery of SCI in rats. The expression of Netrin-1 and DCC decreased significantly at the site of injury after SCI was induced [45]. Compared to the high expression of Netrin-1 caused by the injection of Netrin-1-overexpressing lentivirus into the injured area, the content of DCC in the injured area was lower, which resulted in the production of excessive Netrin-1 that could not bind to DCC, thus hindering the efficacy of Netrin-1 in promoting axonal regeneration. As Netrin-1 can induce and promote axonal growth by binding to its receptor DCC, we injected the Netrin-1-overexpressing lentivirus and DCC-overexpressing lentivirus at the site of SCI at the same time to increase the concentration of both simultaneously, so that none of the molecules of Netrin-1 and DCC remained unbound. The results of RT-PCR and WB assays showed that the simultaneous increase in Netrin-1/DCC increased the expression of GAP43 and SYP in the subacute stage of SCI, and the simultaneous silencing of Netrin-1/DCC decreased the level of expression of GAP43 and SYP. These results indicated that Netrin-1/DCC co-transfection strongly promoted the regeneration of nerve axons in the injured area of the spinal cord.

The Netrin-1/DCC complex has two conditions to promote axonal regeneration: one is the secretion of NTFs that promote regeneration, and the other is the reduction of the effect of factors that inhibit nerve regeneration [46]. Between these two conditions, the inhibitory factors play a greater role, and the NgR1-RhoA-Rock signaling pathway is the main pathway that inhibits nerve axonal regeneration [47]. To further investigate the function and mechanism of the binding of Netrin-1 to DCC, we performed bioinformatics predictions and found that DCC is closely related to NgR1. The results of RT-PCR assays showed that the simultaneous increase in Netrin-1/DCC decreased the expression of factors related to the NgR1 signaling pathway in the subacute phase of SCI. The results also showed that the simultaneous silencing of Netrin-1/DCC increased the expression of these factors. The results of WB assays showed that the simultaneous increase in Netrin-1/DCC decreased the expression of the factors related to the NgR1 signaling pathway in the subacute stage of SCI, and the simultaneous silencing of Netrin-1/DCC increased the expression of these factors. These results indicated that the protective effect of Netrin-1/DCC on rats with injured spinal cords may be related to the promotion of axonal regeneration facilitated by the downregulation of the expression of the NgR1 signaling pathway.

Netrin1/DCC expression is constantly changing after SCI. At present, many studies only report the role of these two genes alone but do not analyze their co-expression. Although many reports have revealed the important role of netrin1 in SCI, there are still many problems with how to rationally and effectively develop these molecular targets, such as the application of netrin1 in clinical diagnosis and treatment. In conclusion, this article has shown that Netrin-1 is involved in multiple pathophysiological processes in SCI. At the cellular level, Netrin-1/DCC has been shown to regulate a range of pathophysiological responses, such as axon regeneration, neuronal apoptosis, glial cell activation, inflammatory response, angiogenesis, and oxidative stress. Therefore, Netrin-1/DCC may be a promising biomarker and target involved in the diagnosis, treatment, and prognosis of SCI. Future research should focus on the detailed mechanism and functional role of Netrin-1/DCC to develop targeted therapies for SCI, and whether this mechanism can be used in the clinical treatment of other neurological injury diseases to better apply it to clinical practice.

## 5 Conclusion

To summarize, in this experimental study conducted with a rat spinal cord T10 level transection model, we found that the binding of Netrin-1 to its receptor DCC promoted axon regeneration and contributed to the recovery of motor function after SCI in rats. Our findings suggested a correlation between Netrin-1/DCC and the NgR1-RhoA-ROCK signaling pathway. Specifically, Netrin-1 promotes axon regeneration by binding to DCC and inhibiting the NgR1-RhoA-ROCK signaling pathway. These results showed a mechanism-based bio-repair strategy for intact SCI injury that can serve as a rehabilitation modality to stimulate axon growth and facilitate functional recovery. Whether Netrin-1 promotes axonal regeneration by inhibiting the NgR1-RhoA-ROCK signaling pathway after binding to its receptor, DCC has not been reported. The study of these mechanisms in this project is of great help to overcome the problem of repair after SCI in clinics as soon as possible, and will open up a new way for clinical treatment of SCI. At the same time, we study whether this mechanism can be used in the treatment of other nerve injury diseases in clinical practice so as to better apply it to clinical practice.

## Supplementary Material

Supplementary Figure
