# Supplementary material for: Lentivirus-mediated overexpression of netrin-1/DCC co-expression promotes axonal regeneration and functional recovery in spinal cord injury via the inhibition of the NgR1-RhoA-ROCK signaling pathway
Source: Transl Neurosci. 2025 Mar 10;16(1):20250365. doi: 10.1515/tnsci-2025-0365 (PMC11909580; doi:10.1515/tnsci-2025-0365)
Supplement: Supplementary Figure [file tnsci-2025-0365-suppl.pdf]

Supplementary material

S1 Successful construction and characterization of Netrin-1 overexpression/low expression lentivirus

A lentivirus-mediated overexpression vector was constructed and transfected into 293T cells for preparation and expression verification of overexpression stable overexpression cell lines (Figure S1a and b). Successfully transfected 293T cells with red fluorescence (Figure S1c), primers for PCR identification of the target gene fragment, positive transformants PCR product size: 109 bp (Figure S2d) In in vitro experiments, we selected the most potent interfering fragment of Netrin-1, RSH089614-LVRH1GP-a (OS718812) fragment, so we directly The fragment was used to package the virus (Figure S2a and b), and after 48 h, the pseudovirus particles were started to be collected, and the fluorescence pictures were collected after inverted fluorescence microscopy before collecting the virus, showing that: green fluorescence was visible in 293T cells in the fluorescence pictures. It indicates that the cells packed virus successfully (Figure S2c). In addition, according to RT-qPCR analysis, Netrin-1 levels were significantly decreased in the Netrin-1-si group compared to the NC group (Figure S2d). the production of Netrin-1-si virus effectively transfected PC12 cells and stably expressed the marker RNA in vitro.

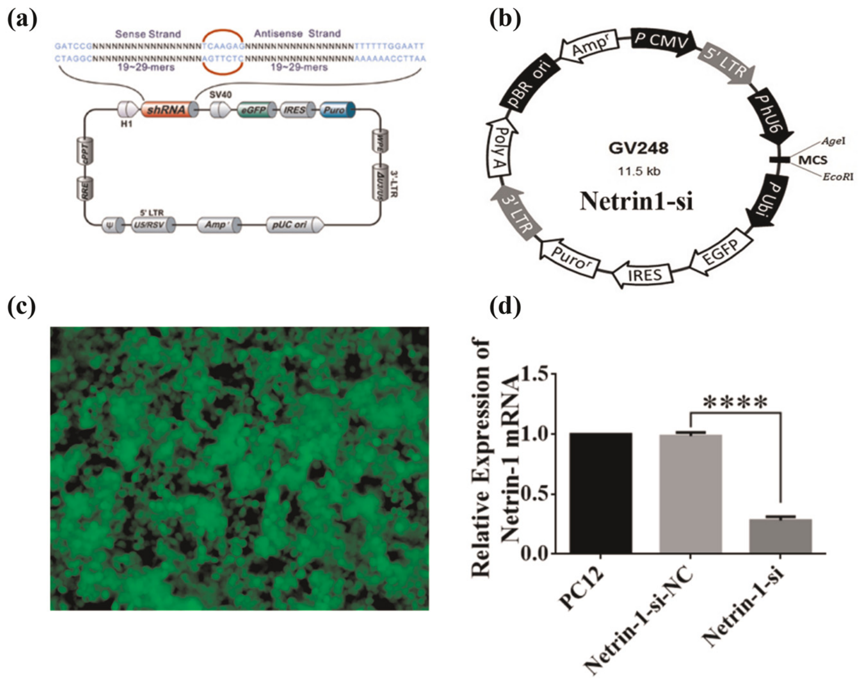

Figure S1: Lentivirus-mediated netrin-1 shRNA recombinant construction. (a) Screening vector for fragment a. Vector name is psi-LVRH1GP, 5′ cloning site. b. amHI 3′ cloning site: EcoRI. (b) Lentiviral vector map, vector name is GV248; component sequence: hU6-MCS-Ubiquitin-EGFP-IRES-puromycin; control insert sequence. TTCTCCGAACGTGTCACGT; control number: CON077; vector map: http://www.genechem.com.cn/service/index.php?ac=gene&at=vector_search&keyword=GV248. (c) 293T cells were successfully transfected with lentivirus, emitting green fluorescence, picture is 100×. (d) RT-PCR detection of netrin-1 expression in PC12 cells. ****$P < 0.0001$.

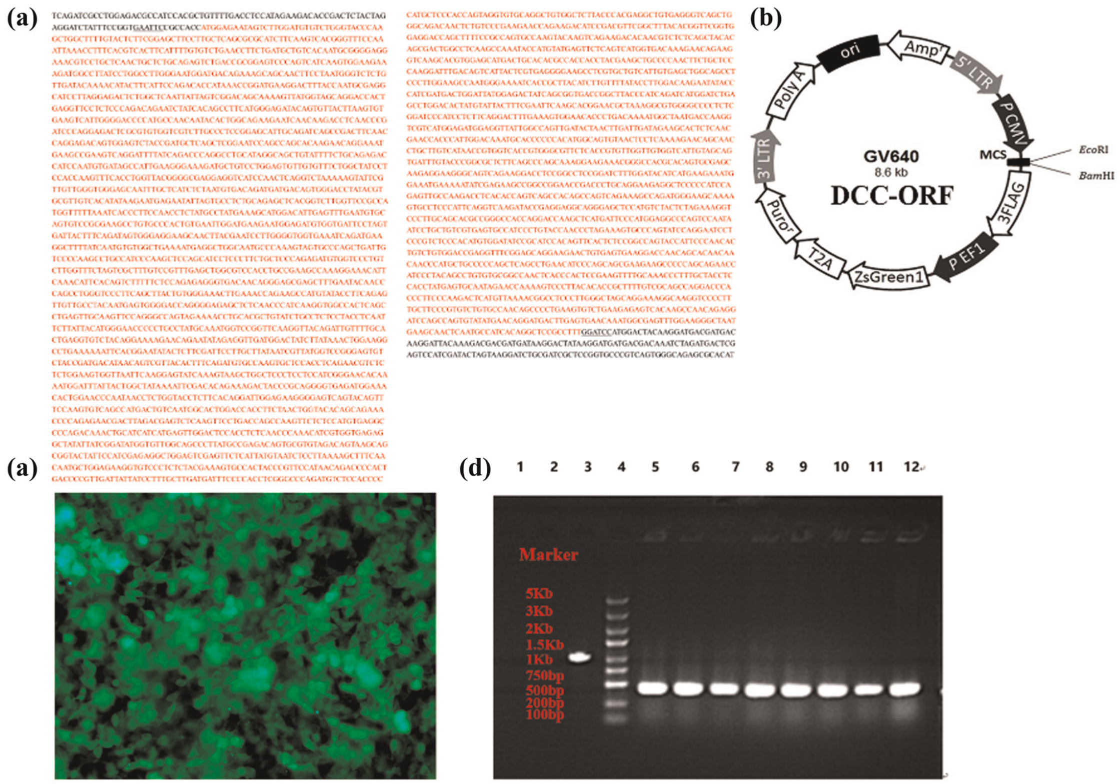

Figure S2: Construction and identification of DCC overexpression lentivirus. (a) Sequencing comparison results of positive clones of DCC with AgeI digest site underlined at 5′ end, AgeI digest site underlined at 3′ end, and target sequence marked in red. (b) Vector map, vector name GV640; component order: CMV-MCS-3FLAG-EF1a- ZsGreen1-T2A-puromycin; cloning site: EcoRI / BamHI; control number: CON470; vector map: http://www.genechem.com.cn/index/supports/tool_search.html?keywords=GV640 Vector instructions can be downloaded. (c) Successful lentiviral transfection in 293T cells with green fluorescence, image is 100×. (d) PCR electrophoresis. 1#: negative control (ddH_2O); 2#: negative control (empty self-linked control); 3#: positive control (GAPDH); 4#: Marker in the order of 5 kb, 3 kb, 2 kb, 1.5 kb from top to bottom, 1 Kb, 750 bp, 500 bp, 250 bp, 100 bp; 5–12#: transformants 1–8.

S2 Successful construction and characterization of DCC overexpression/low expression lentivirus

We successfully constructed a lentivirus-mediated overexpression vector and transfected into 293T cells for preparation and expression verification of overexpression stable overexpression cell lines (Figure S2a and b). Successfully transfected 293T cells with green fluorescence (Figure S2c), primers for PCR to identify the target gene fragment, positive transformants PCR product size: 415 bp (Figure S2d). In the in vitro part of the experiment, we selected the most potent DCC interfering fragment: RSH049164-LVRH1MP-b (OS682785) fragment, so we used the screened fragment to package the virus (Figure S3a and b), and after 48 h, started collecting pseudovirus particles, and before collecting the virus after inverted fluorescence microscopy to collect fluorescence pictures, showing: fluorescence pictures of 293T cells were visible as red fluorescence. This indicates that the cells were successfully packed with virus (Figure S3c). By RT-qPCR analysis, the DCC level was significantly decreased in the DCC-si group compared to the NC group (Figure S3d). the production of DCC-si virus effectively transfected PC12 cells and stably expressed the marker RNA in vitro.

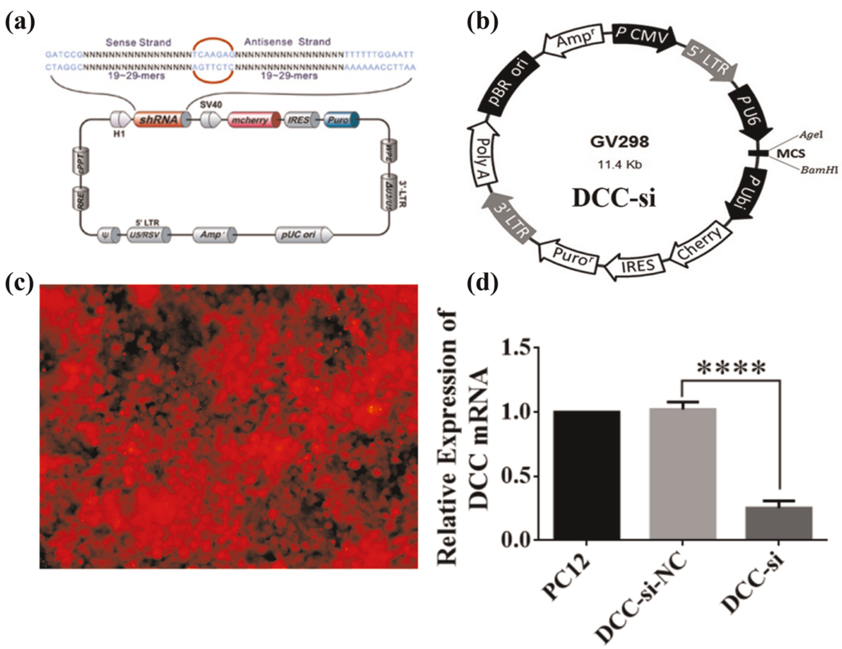

Figure S3: Lentivirus-mediated recombinant construction of DCC shRNA. (a) Screening vector for fragment a. Vector name is psi-LVRH1MP, 5′ cloning site: b.amHI 3′ cloning site: EcoRI. (b) Lentiviral vector map, vector name is GV298; component sequence: U6-MCS-Ubiquitin-Cherry-IRES-puromycin; control insert sequence. TTCTCCGAACGTGTCACGT; control number: CON207; vector map: http://www.genechem.com.cn/service/index.php?ac=gene&at=vector_search&keyword=GV298. (c) 293T cells were successfully transfected with lentivirus, emitting red fluorescence, picture is 100 times. (d) RT-qPCR to detect the expression of DCC in 293T cells. *P < 0.05, **P < 0.01, ***P < 0.001 vs CSHCTR001-LVRH1GP (control group).
